# Src- and Abl-family kinases activate spleen tyrosine kinase to maximize phagocytosis and *Leishmania* infection

**DOI:** 10.1242/jcs.260809

**Published:** 2023-07-28

**Authors:** Imran Ullah, Umaru Barrie, Rebecca M. Kernen, Emily T. Mamula, Francis Tho Huu Khuong, Laela M. Booshehri, Emma L. Rhodes, James M. Bradford, Arani Datta, Dawn M. Wetzel

**Affiliations:** ^1^ Department of Pediatrics, University of Texas Southwestern Medical Center, 5323 Harry Hines Blvd, Dallas, TX 75390, USA; ^2^ Department of Biochemistry, University of Texas Southwestern Medical Center, 5323 Harry Hines Blvd, Dallas, TX 75390, USA; ^3^ Medical Scientist Training Program, University of Texas Southwestern Medical Center, 5323 Harry Hines Blvd, Dallas, TX 75390, USA; ^4^ Emergency Medicine Residency Program, University of Texas Southwestern Medical Center, 5323 Harry Hines Blvd, Dallas, TX 75390, USA

**Keywords:** *Leishmania*, Kinase, Pathogen, Parasite, Macrophage

## Abstract

*Leishmania* spp. are obligate intracellular parasites that must be internalized by phagocytic cells to evade immune responses and cause disease. The uptake of both *Leishmania* promastigotes (insect-stage parasites) and amastigotes (proliferative-stage parasites in humans and mice) by phagocytes is thought to be mainly host cell driven, not parasite driven. Our previous work indicates that host Src- and Abl-family kinases facilitate *Leishmania* entry into macrophages and pathogenesis in murine cutaneous leishmaniasis. Here, we demonstrate that host spleen tyrosine kinase (SYK) is required for efficient uptake of *Leishmania* promastigotes and amastigotes. A Src-family kinase–Abl-family kinase–SYK signaling cascade induces *Leishmania* amastigote internalization. Finally, lesion size and parasite burden during *Leishmania* infection is significantly decreased in mice lacking SYK in monocytes or by treatment with the SYK inhibitor entospletinib. In summary, SYK is required for maximal *Leishmania* uptake by macrophages and disease in mice. Our results suggest potential for treating leishmaniasis using host cell-directed agents.

## Teaser

**Summary:** Activation of spleen tyrosine kinase by Src- and Abl-family kinases is required for maximal *Leishmania* uptake by macrophages and disease in a mouse model of cutaneous leishmaniasis.

## INTRODUCTION

Approximately 350 million people worldwide are at risk of the disfiguring cutaneous or fatal visceral forms of leishmaniasis, which is a neglected tropical disease (https://www.who.int/news-room/fact-sheets/detail/leishmaniasis). The drugs currently used to treat leishmaniasis have poor efficacy and/or are highly toxic to human hosts; therefore, new antiparasitics are urgently needed (Wetzel and Phillips, 2018). Leishmaniasis results from infection by obligate intracellular parasites of the *Leishmania* genus. During its life cycle, *Leishmania* alternates between promastigotes, which are found in sand fly vectors, and amastigotes, which are found in vertebrate hosts such as humans, dogs and rodents. In order for infection to be established in humans after a sand fly bite, phagocytic cells [for example, neutrophils or macrophages (Mφs)] must ingest promastigotes, using a process that is thought to be primarily driven by the host cell (Ueno and Wilson, 2012). The parasites survive in acidic phagolysosomes, where they differentiate into amastigotes and multiply. If amastigotes exit this compartment, they must be taken up again by phagocytes to survive within their vertebrate hosts (Ueno and Wilson, 2012). *Leishmania* parasites that remain outside of phagocytic cells are thought to either die or be killed by the host immune system.

Multiple protein receptors on the surfaces of phagocytic cells have been implicated in the process of *Leishmania* uptake. For example, promastigotes bind the complement receptor CR3 [which comprises integrin subunit α M (ITGAM) and integrin β2 (ITGB2)]; this process is enhanced when parasites are opsonized (coated) with the terminal component of complement, C3bi (also known as iC3b, derived from complement C3) (Mosser et al., 1992; Puentes et al., 1988; Russell and Wright, 1988). Amastigotes are opsonized with immunoglobulin G (IgG) and bind the Fc receptor (FcR) subclass FcγR (Guy and Belosevic, 1993), which is required for a productive *in vivo* infection (Kima et al., 2000). After *Leishmania* binds cell surface receptors, actin-rich phagocytic cups form and surround the parasites, allowing them to be internalized (Lodge and Descoteaux, 2008). However, the host cell signaling process that drives *Leishmania* uptake has not been well described.

Previously, we have implicated host Src-family kinases (SFKs) as well as Abl-family kinases in the process of *Leishmania amazonensis* uptake by Mφs (Wetzel et al., 2016). The SFKs, and specifically Hck, Fgr and Lyn, are non-receptor tyrosine kinases that are known to participate in IgG-mediated phagocytosis (Fitzer-Attas et al., 2000) after pathogens bind to the FcγR (Wu et al., 2001). Using SFK inhibitors, such as SU6656, and primary Mφs lacking Hck, Fgr and Lyn, we have previously demonstrated that SFKs are necessary for maximal amastigote uptake by Mφs, as well as for disease in a mouse model of cutaneous leishmaniasis (Wetzel et al., 2016). SFKs bind and phosphorylate another set of non-receptor tyrosine kinases, the Abl-family kinases Abl (Abl1) and Arg (Abl2) (Mader et al., 2011; Plattner et al., 2004; Tanis et al., 2003; Bradley and Koleske, 2009). We have shown that Abl and Arg facilitate complementary activities during phagocytosis and *Leishmania* uptake by Mφs (Wetzel et al., 2012). Specifically, Mφs isolated from mice lacking Arg (*Arg^−/−^* Mφ) exhibit reduced phagocytosis of IgG-coated beads or amastigotes but have no defects in the uptake of C3bi-coated beads or promastigotes (Wetzel et al., 2012). Conversely, Mφs from mice lacking Abl (*Abl^−/−^* Mφ) show defects in the uptake of C3bi-coated beads and promastigotes but have no defects in the internalization of IgG-coated beads or amastigotes (Wetzel et al., 2012). Mφs lacking both Abl-family kinases have no deficiencies in C3bi- or IgG-mediated uptake beyond that seen for Mφs lacking the single relevant kinase (Wetzel et al., 2012, 2016). Mice lacking Abl or Arg, or treated with the Abl-family kinase inhibitor imatinib, have smaller lesions and lower parasite burden in a cutaneous mouse model of leishmaniasis (Wetzel et al., 2012). Finally, treatment of *Leishmania*-infected mice with bosutinib, which inhibits both Abl-family kinases and SFKs, leads to greater decreases in lesion size and parasite burden than are seen upon inhibition of either kinase family alone (Wetzel et al., 2016).

Spleen tyrosine kinase (SYK) is a cytoplasmic tyrosine kinase with ten tyrosines that can be phosphorylated to participate in signal transduction from immune receptors such as FcγR (Furlong et al., 1997). Mφs derived from mice conditionally lacking SYK in immune cells are defective in several FcγR-induced signaling events (Bonnerot et al., 1998; Bradshaw, 2010; Lowell, 2011). These Mφs also have deficits in IgG-mediated phagocytosis; they form actin-rich phagocytic cups but cannot close them around pathogens (Crowley et al., 1997). However, these Mφs respond normally to lipopolysaccharide, indicating that there are no deficiencies in activation (Crowley et al., 1997). Some laboratories have suggested that SYK inhibition does not affect C3bi-mediated phagocytosis (Kiefer et al., 1998), but others have shown significant defects in this process (Tohyama and Yamamura, 2006; Walbaum et al., 2021). There do not appear to be SYK homologs in *Leishmania* (Baker et al., 2021; Parsons et al., 2005).

Previous studies suggest that kinases implicated in *Leishmania* internalization might also modulate SYK-mediated processes in related biological systems. For example, Mφs derived from mice deficient for Hck, Fgr and Lyn exhibit decreased SYK activation when FcγR is crosslinked by antibodies (Crowley et al., 1997). In addition, loss of Abl and/or Arg activity limits SYK phosphorylation after FcγR crosslinking with antibodies, and Arg kinase phosphorylates SYK in kinase assays with purified proteins (Greuber and Pendergast, 2012). Based on these previous findings, we hypothesized that host cell SYK would be activated by SFKs and Abl-family kinases during the uptake of *Leishmania* by Mφs, and that SYK activity would be required for pathogenesis in a mouse model of leishmaniasis.

Here, we demonstrate that host cell SYK facilitates both C3bi- and IgG-mediated phagocytosis and promotes the internalization of *Leishmania* promastigotes and amastigotes. An SFK–Abl-family kinase–SYK pathway is required for maximal internalization of *Leishmania* amastigotes by Mφs. In addition, mice lacking SYK in monocytes, as well as mice treated with the SYK kinase inhibitor entospletinib, have significantly reduced lesion severity and parasite burden in a mouse model of cutaneous *Leishmania* infection. Our results provide proof-of-concept that leishmaniasis could potentially be treated with agents directed at host cell kinases such as SYK.

## RESULTS

### Entospletinib decreases complement- and immunoglobulin-mediated phagocytosis

Previously, we have shown that SFK inhibitors and Abl-family kinase inhibitors decrease the uptake of *Leishmania* parasites (Wetzel et al., 2012, 2016). Others have suggested that signaling during phagocytosis by these two kinase families can be relayed to SYK (Crowley et al., 1997; Greuber and Pendergast, 2012). Therefore, we chose to explore the role of SYK in phagocytic model systems and *Leishmania* uptake by Mφs.

Entospletinib is a SYK inhibitor that has a half-maximal inhibitory concentration (IC_50_) of 7.7 nM in purified kinase assays (Ramanathan et al., 2017). Given its recent favorable results in a human phase Ib/II trial for acute myeloid leukemia (Walker et al., 2020), it is an attractive tool for further study; however, its effects on phagocytosis had not been documented previously. For this reason, we first determined whether entospletinib treatment of phagocytic cells affected immunoglobulin-mediated phagocytosis. We treated RAW 264.7 cells, a murine Mφ-like cell line, with increasing concentrations of entospletinib or DMSO for 2 h and incubated them for 30 min with beads that had been coated with IgG. We found that entospletinib, in a concentration-dependent manner, decreased the uptake of IgG-coated beads, as demonstrated by the phagocytic index (PI), which was calculated as the number of internalized beads per 100 Mφs (Fig. S1A). Based on this titration, we selected a concentration of 1 µM for further studies.

**Fig. 1.**
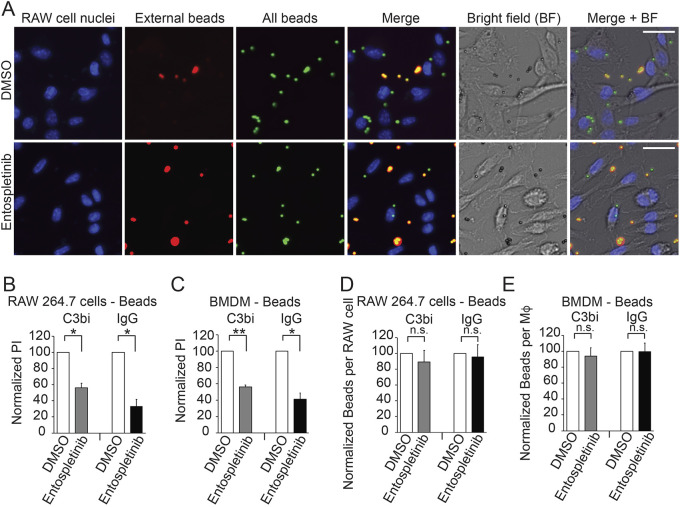
**The SYK inhibitor entospletinib decreases C3bi- and IgG-mediated phagocytosis.** Mφs were treated with 1 µM entospletinib or DMSO for 2 h and incubated with C3bi- or IgG-coated beads for 30 min. A multi-colored immunofluorescence assay was used to distinguish between extracellular (red and green) and intracellular (green only) beads (see Materials and Methods for details). Nuclei are labeled with Hoechst 33258. (A) Images of C3bi-opsonized bead uptake by RAW 264.7 cells treated with DMSO (top) or entospletinib (bottom). Scale bars: 10 µm. In the top panel, there are portions of 13 Mφ nuclei visible, with 22 total beads and seven external beads (i.e. 15 internalized beads), suggesting a PI of 115. In the bottom panel, 11 Mφ nuclei are seen, with portions of 23 total beads and 19 extracellular beads (i.e. four intracellular beads), suggesting a PI of 36. (B) Quantification of the effects of entospletinib on C3bi-coated and IgG-coated bead uptake by RAW 264.7 cells. Shown is the mean±s.e. PI for RAW 264.7 cells incubated in 1 µM entospletinib and allowed to take up C3bi-coated (left) or IgG-coated beads (right), normalized to RAW 264.7 cells incubated in an equivalent volume of DMSO and the same number of beads (shown as a PI of 100). (C) Quantification of the effect of entospletinib on C3bi-coated and IgG-coated bead uptake by BMDMs. Shown is the mean±s.e. PI, calculated as described in B. (D,E) Entospletinib does not affect the total number of C3bi- and IgG-coated beads bound to (D) RAW 264.7 cells and (E) BMDMs. Shown is the mean±s.e. total number of beads per Mφ after Mφs were incubated in 1 µM entospletinib and allowed to take up C3bi-coated (left) or IgG-coated beads (right), normalized to Mφs incubated in an equivalent volume of DMSO and beads. For B–E, *n*=3 separate experiments. *P<0.05; **P<0.01; ns, not significant (two-tailed one-sample *t*-test).

Whether SYK plays a role in C3bi-mediated phagocytosis has been a subject of debate (Kiefer et al., 1998; Tohyama and Yamamura, 2006; Walbaum et al., 2021). To determine whether entospletinib treatment of host cells affected C3bi- and IgG-mediated phagocytosis, we treated RAW 264.7 cells with 1 µM entospletinib or DMSO for 2 h and incubated them for 30 min with beads that had been coated with C3bi or IgG. We found that 1 µM entospletinib decreased both C3bi-coated and IgG-coated bead uptake by RAW 264.7 cells by 44±6% and 67±8% (mean±s.e.), respectively (Fig. 1A,B). Since RAW 264.7 cells are a cancer cell line that has been virally transformed, we confirmed these results using primary cells: bone marrow-derived Mφs (BMDMs) isolated from mouse tibias/femurs (Fig. 1C). The decreases in C3bi- and IgG-mediated phagocytosis due to entospletinib treatment specifically resulted from defects in internalization, as entospletinib did not affect the total (internal plus external) number of C3bi- and IgG-coated beads normalized to the number of RAW 264.7 cells (Fig. 1D) or BMDMs (Fig. 1E).

### SYK inhibition decreases *Leishmania* promastigote and amastigote uptake by Mφs

**Fig. 2.**
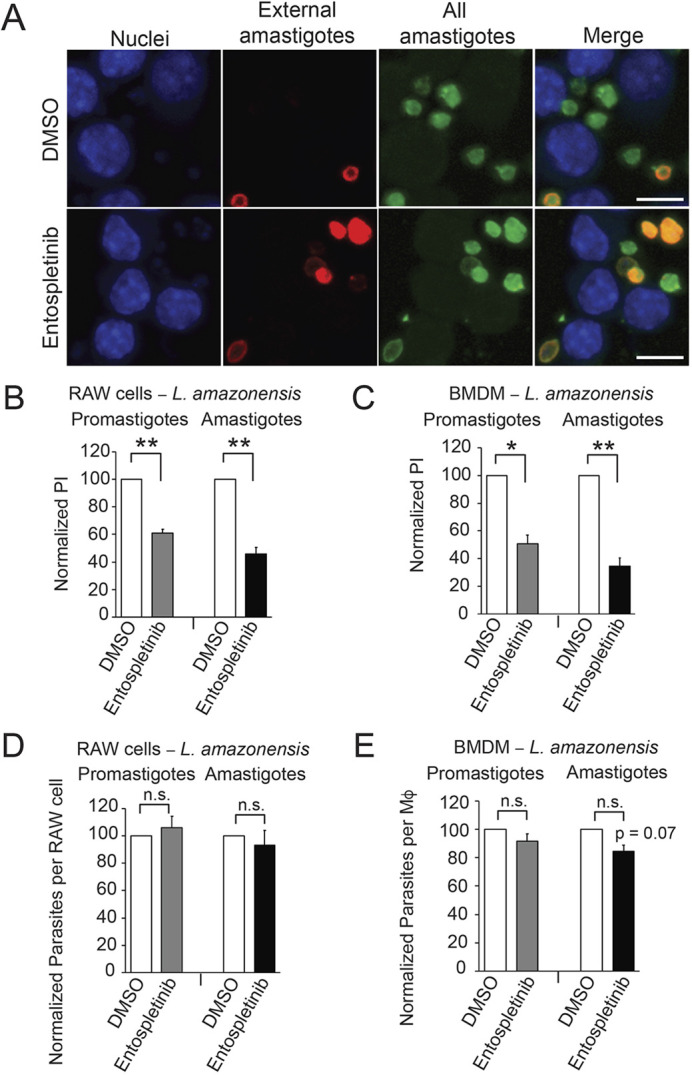
**The SYK inhibitor entospletinib decreases *Leishmania* uptake.** Mφs were treated with 1 µM entospletinib or DMSO for 2 h and incubated with C3bi-coated *L. amazonensis* promastigotes or IgG-coated *L. amazonensis* amastigotes for 30 min. Immunofluorescence was used to distinguish between extracellular (red and green) and intracellular (green only) promastigotes or amastigotes. Mφ nuclei are labeled with Hoechst 33258. (A) Image of anti-P8 IgG-opsonized amastigote uptake by RAW 264.7 cells treated with DMSO (top) or entospletinib (bottom). Scale bars: 5 µm. In the top panel there are portions of five Mφ nuclei visible, with seven total amastigotes and two external amastigotes (i.e. five internalized amastigotes), suggesting a PI of 100. In the bottom panel, four Mφ nuclei are seen, with seven total amastigotes and five extracellular amastigotes (i.e. two intracellular amastigotes), suggesting a PI of 50. (B,C) Quantification of the effects of entospletinib on promastigote or amastigote uptake by (B) RAW 264.7 cells and (C) BMDMs. Shown is the mean±s.e. PI for Mφs incubated in 1 µM entospletinib and allowed to take up promastigotes (left) or amastigotes (right), normalized to Mφs incubated in an equivalent volume of DMSO and the same number of parasites. (D,E) Entospletinib does not affect the total number of promastigotes and amastigotes bound to (D) RAW 264.7 cells and (E) BMDMs. Shown is the mean±s.e. total number of parasites per Mφ after Mφs were incubated in 1 µM entospletinib and allowed to take up promastigotes (left) or amastigotes (right), normalized to Mφs incubated in an equivalent volume of DMSO and the same number of parasites. For B–E, *n*=3 separate experiments. *P<0.05; **P<0.01; ns, not significant (two-tailed one-sample *t*-test).

We can grow both promastigotes and amastigotes of *L. amazonensis* without mammalian host cells (axenic cultures), using a supplemented growth medium at pH 7.2 for promastigotes or pH 5.0 for amastigotes (Hodgkinson et al., 1996). To test whether entospletinib treatment also affected the internalization of promastigotes and axenic amastigotes, we again pre-treated Mφs with 1 µM entospletinib or DMSO, but then incubated them with C3bi-coated promastigotes or IgG-coated axenic amastigotes of *L. amazonensis.* Representative images of amastigote uptake with and without entospletinib are shown in Fig. 2A. We found that 1 µM entospletinib decreased promastigote and amastigote uptake by RAW 264.7 cells (by 39±3% and 54±5%, respectively; mean±s.e.; Fig. 2B) and BMDMs (by 49±6% and 66±6%, respectively; Fig. 2C). Titration of entospletinib demonstrated that promastigote uptake and amastigote uptake by RAW 264.7 cells was decreased in a concentration-dependent manner (Fig. S1B and C, respectively). Entospletinib did not affect the total number of promastigotes or amastigotes that bound to RAW 264.7 cells (Fig. 2D) or BMDMs (Fig. 2E).

To ensure that entospletinib did not have deleterious effects on *L. amazonensis* amastigotes, we incubated axenic amastigotes with entospletinib for 72 h and assessed parasite survival using alamarBlue (Ullah et al., 2020). The half-maximal effective concentration (EC_50_) of entospletinib against axenic amastigotes in this assay was greater than 20 µM. The 50% cytotoxic concentration (CC_50_) for RAW 264.7 cells over 72 h was 3.3±1.2 µM (mean±s.e.; three biological replicates performed; see representative log concentration response curves in Fig. S1D). These studies indicated that entospletinib did not kill either *Leishmania* parasites or host cells over the time period and concentrations studied in these uptake assays. To test whether entospletinib affected the intracellular survival of *L. amazonensis* amastigotes post-internalization, we engineered *L. amazonensis* parasites that expressed mNeonGreen (see Materials and Methods) and allowed these amastigotes to be taken up by RAW 264.7 cells. After 24 h, we added DMSO or 0.5 µM entospletinib (a concentration where effects on host cells were minimal) for 72 h. We found no apparent defects in the ability of amastigotes to survive within 0.5 µM entospletinib-treated RAW 264.7 cells compared to survival within DMSO-treated RAW 264.7 cells over 72 h (Fig. S1E).

We next explored the mechanism by which entospletinib affected the internalization of *Leishmania* parasites. First, we determined that extending uptake over longer periods (up to 3 h) did not enable treated RAW 264.7 cells to overcome entospletinib-mediated defects in *Leishmania* internalization (promastigotes shown in Fig. S1F; amastigotes shown in Fig. S1G). To more specifically characterize the effects of entospletinib on phagocytic cup formation, we performed an uptake assay with amastigotes as described above, then incubated samples with fluorescently labeled (far-red) phalloidin. As has previously been described for SYK inhibition (Crowley et al., 1997), using confocal microscopy, we found that phagocytic cups seemed unable to close in entospletinib-treated RAW 264.7 cells (Fig. S2), particularly since there was no difference between the number of cups seen for DMSO- and entospletinib-treated Mφs (Fig. S3A). In addition, actin staining in phagocytic cups was brighter on average in entospletinib-treated RAW 264.7 cells than in DMSO-treated RAW 264.7 cells (Fig. S3B), suggesting a direct effect on actin during phagocytic cup formation.

### Host cell SYK is required for efficient phagocytosis and *Leishmania* uptake by Mφs

**Fig. 3.**
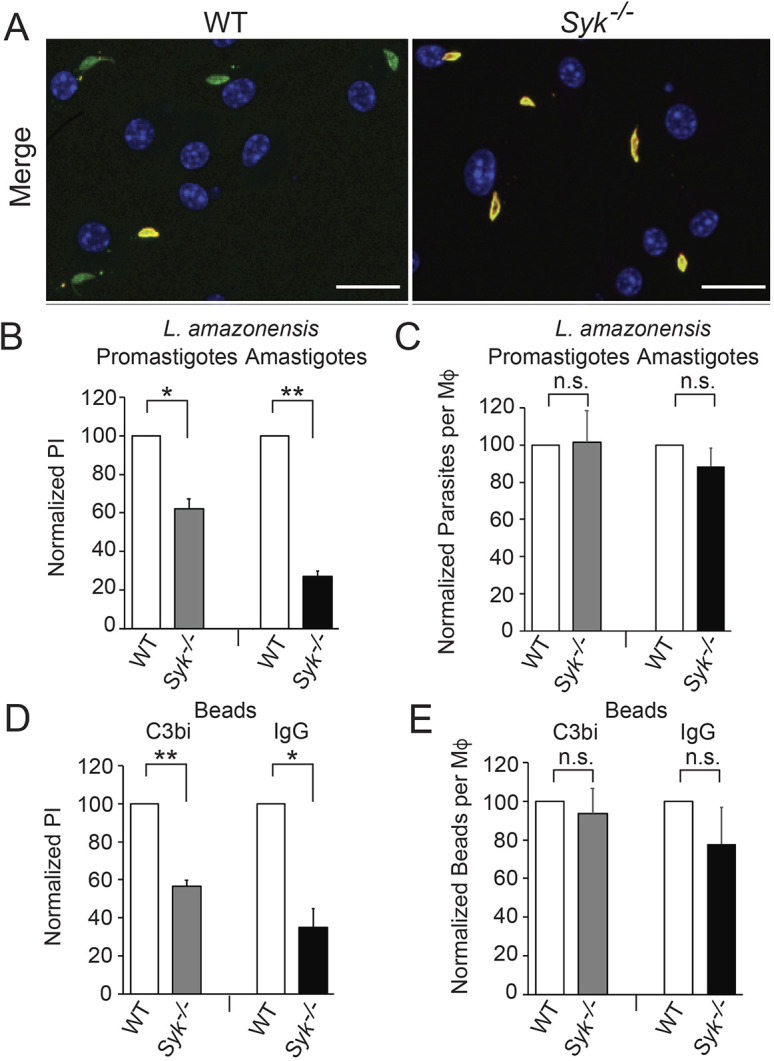
**BMDMs lacking SYK exhibit defects in C3bi- and IgG-mediated phagocytosis and *L. amazonensis* uptake.** (A) Representative merged images of promastigote uptake by WT BMDMs (left) or *Syk^flox/flox^* LysM Cre+ BMDM (*Syk^−/−^* BMDMs, right), using the methodology described in Fig. 2. Scale bars: 10 µm. On the left there are eight full Mφ nuclei visible, with five total promastigotes and four internalized promastigotes, suggesting a PI of 50. On the right, seven Mφ nuclei are seen, with five total promastigotes and no intracellular promastigotes. (B) *Syk^−/−^* BMDMs exhibit defects in promastigote and amastigote uptake. BMDMs were incubated with *L. amazonensis* promastigotes or amastigotes as in Fig. 2. Graph shows the mean±s.e. PI for promastigotes and amastigotes for *Syk^−/−^* BMDMs, normalized to that of WT BMDMs, using the methodology described in Fig. 2. (C) Mean±s.e. normalized total number of promastigotes or amastigotes bound to *Syk^−/−^* BMDMs, normalized to WT BMDMs. (D) *Syk^−/−^* BMDMs are less able to undergo C3bi- and IgG-mediated phagocytosis. BMDMs were incubated with C3bi- or IgG-coated beads, as in Fig. 1. Shown is the mean±s.e. PI for beads taken up by *Syk^−/−^* BMDMs, normalized to that of WT BMDMs (using the methodology described in Fig. 1). (E) Mean±s.e. normalized total number of C3bi- or IgG-coated beads bound to *Syk^−/−^* BMDMs, normalized to WT BMDMs. *n*=3 separate experiments. *P<0.05; **P<0.01; n.s., not significant (two-tailed one sample *t*-test).

Given that kinase inhibitors can potentially have off-target effects, and could also be acting upon either parasites or host cells, we next specifically tested the role of host cell SYK during *Leishmania* uptake by Mφs. Using BMDMs isolated from mice lacking SYK in the monocyte lineage (*Syk^flox/flox^* LysM Cre+; abbreviated as *Syk^−/−^* when discussing isolated BMDMs), we found that *Syk^−/−^* BMDMs demonstrated decreases in promastigote (Fig. 3A,B) and amastigote uptake (Fig. 3B), by 38±5% and 73±3% (mean±s.e.), respectively. The total number of promastigotes or amastigotes bound to BMDMs was not affected by loss of SYK (Fig. 3C). *Syk^−/−^* BMDMs also exhibited defects in C3bi- and IgG-mediated phagocytosis of coated beads (which were decreased by 43±3% and 65±10%, respectively; Fig. 3D,E).

### *Leishmania* stimulates SFK–Arg–SYK signaling in Mφs to stimulate uptake

We next explored whether *L. amazonensis* employs signaling by the adhesion molecule PSGL-1 (CD162, also known as SELPLG) to facilitate uptake. PSGL-1 has not been shown to be a receptor for *Leishmania* uptake, but it is known to activate SYK signaling via interactions with FcγR (Zarbock et al., 2008). We studied both of these possibilities using an antibody known to block PSGL-1 signaling (Zanardo et al., 2004), which should also prevent *Leishmania* from directly interacting with PSGL-1. We found that this antibody did not affect *Leishmania* internalization (Fig. S3C). In addition, entospletinib still prevented *Leishmania* uptake when added alongside the antibody (Fig. S3D).

**Fig. 4.**
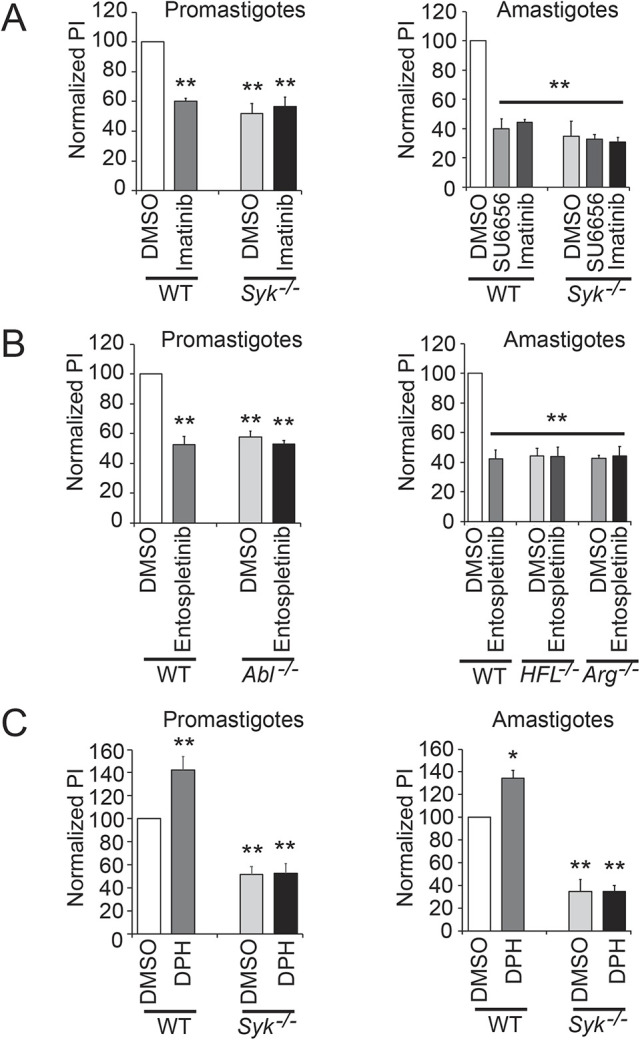
**A SFK–Abl/Arg–SYK signaling pathway facilitates *Leishmania* uptake.** (A) Treating *Syk^−/−^* BMDMs with SU6656 or imatinib does not further diminish *Leishmania* promastigote (left) or amastigote (right) uptake. (B) Entospletinib has no effect on promastigote uptake by BMDMs lacking Abl (left) or amastigote uptake by BMDMs lacking Hck, Fgr and Lyn (*HFL^−/−^*), or Arg kinases (right). (C) The Arg/Abl activator DPH does not rescue *Leishmania* promastigote (left) or amastigote uptake (right) in *Syk^−/−^* BMDMs. For A–C, BMDMs of the genotypes shown were incubated with 0.1% DMSO, 1 µM SU6656, 3.3 µM imatinib, 1 µM entospletinib or 50 nM DPH for 2 h, as indicated, and allowed to take up promastigotes or amastigotes for 30 min. PIs were calculated and values were normalized to WT, DMSO-treated BMDMs (100%). Mean±s.e. shown. *n*=3 separate biological experiments. *P<0.05; **P<0.01 (two-way ANOVA with Tukey's post hoc test, compared to WT DMSO-treated Mφs).

SFKs and Abl-family kinases have been shown to partly signal through SYK in previous studies (Greuber and Pendergast, 2012). We have shown that Hck, Fgr and Lyn specifically activate Arg to facilitate amastigote uptake by Mφs (Wetzel et al., 2012, 2016). SFKs are not required for C3bi-mediated phagocytosis in our model (Wetzel et al., 2016). Abl, but not SFKs or Arg, is required for efficient promastigote uptake (Wetzel et al., 2012, 2016). Therefore, we hypothesized that an FcγR–SFK–Arg–SYK signaling pathway would be required for amastigote uptake, and a CR3–Abl–SYK signaling pathway would be responsible for promastigote uptake. To test this hypothesis, we performed experiments using combinations of kinase inhibitors and activators (Mohamed et al., 2022). Treating *Syk^−/−^* BMDMs with imatinib to inhibit Abl-family kinases did not further decrease *Leishmania* promastigote uptake, and similarly, treating *Syk^−/−^* BMDMs with SU6656 to inhibit SFKs or with imatinib to inhibit Abl-family kinases did not further diminish *Leishmania* amastigote uptake (Fig. 4A). Adding entospletinib neither further decreased promastigote uptake by BMDMs lacking Abl (*Abl^flox/flox^* LysM Cre+ BMDMs; referred to here as *Abl^−/−^*) nor further impaired amastigote uptake by BMDMs lacking Hck, Fgr and Lyn (*HFL^−/−^* BMDMs), or Arg (*Arg^−/−^* BMDMs) kinases (Fig. 4B). In addition, the Arg/Abl activator DPH did not rescue the uptake of *Leishmania* promastigotes or amastigotes by *Syk^−/−^* BMDMs (Fig. 4C).

**Fig. 5.**
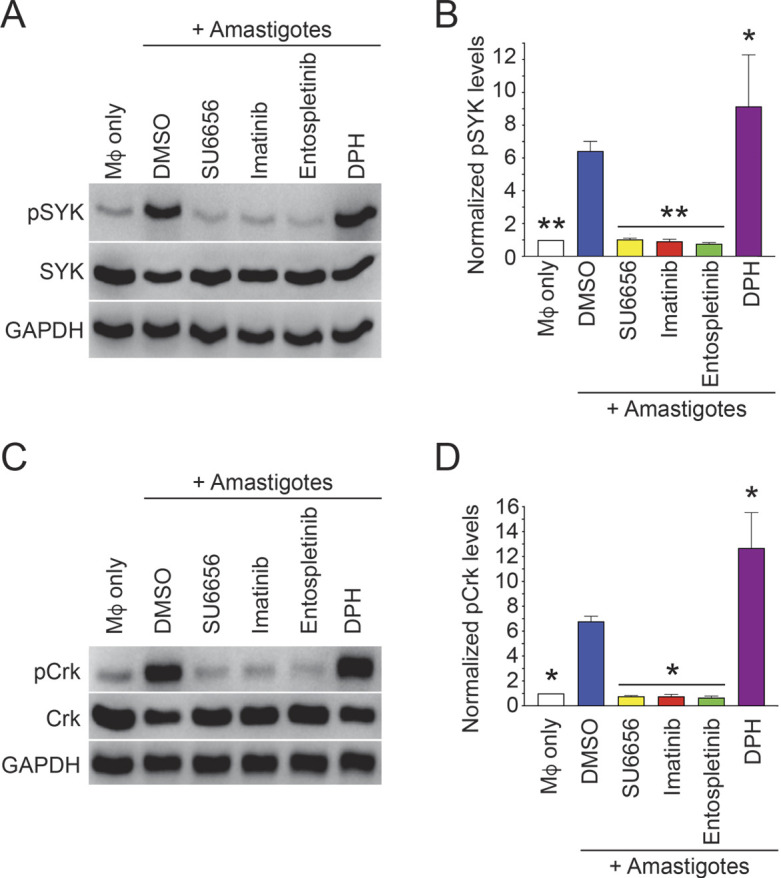
**SYK is activated by SFKs and Arg, and phosphorylates downstream effectors during amastigote uptake.** (A,B) SYK phosphorylation is decreased by inhibition of SFKs or Abl-family kinases, and is increased by activating Abl-family kinases. RAW 264.7 cells treated with DMSO, 3.3 µM SU6656, 3.3 µM imatinib, 1 µM entospletinib or 50 nM DPH for 2 h were incubated with IgG-coated amastigotes (+amastigotes) for 15 min before processing for immunoblotting. (A) Representative immunoblot of pSYK (top) and total SYK (middle) in DMSO-treated RAW 264.7 cells (±amastigotes) and amastigote-stimulated SU6656-, imatinib-, entospletinib- or DPH-treated RAW 264.7 cells. GAPDH (bottom) is used as an additional loading control. (B) Graph presents relative pSYK levels, normalized to SYK levels, among RAW 264.7 cell categories shown in A. pSYK/SYK levels for Mφs only (i.e. no amastigotes) were normalized to 1. (C,D) Phosphorylation of the SFK, Abl and Arg substrate CrkII (pCrk) induced upon amastigote uptake is decreased in entospletinib-treated Mφs. (C) Representative immunoblot of pCrk (top) and total CrkII (middle) in DMSO-treated RAW 264.7 cells (with or without amastigotes) and amastigote-stimulated SU6656-, imatinib-, entospletinib- or DPH-treated RAW 264.7 cells. GAPDH is used as an additional loading control. (D) Relative levels for pCrk, normalized to total CrkII, graphed as described in B. For all categories, *n*=4 separate biological experiments; mean±s.d. shown. *P<0.05, **P<0.01 by two-way ANOVA with Tukey's post hoc test, compared to pSYK (B) or pCrk (D) levels in WT DMSO-treated Mφs incubated with amastigotes.

We then investigated whether SFKs and Abl-family kinases were essential for SYK activation during amastigote uptake. We chose to focus on amastigotes as the more biologically relevant life cycle stage in human infection. We allowed Mφs to begin internalizing amastigotes and performed immunoblots for phosphorylated SYK (pSYK), normalized to total SYK levels. We found that pSYK levels were decreased after inhibition of SFKs (using SU6656) or Abl-family kinases (using imatinib), whereas activating Abl-family kinases using DPH increased SYK phosphorylation (Fig. 5A,B; see Fig. S4 for full western blot images and additional examples). Previously, we have shown that SFKs and Abl-family kinases are required for effective phosphorylation of their substrate CrkII (an isoform of Crk) during amastigote uptake by Mφs (Wetzel et al., 2012, 2016). To determine whether SYK was required for this phosphorylation event, we probed for induction of CrkII phosphorylation (pCrk) upon amastigote uptake by entospletinib-treated Mφs. We found lower levels of pCrk in entospletinib-treated Mφs undergoing amastigote uptake when compared to levels in DMSO controls (Fig. 5C,D; Fig. S4).

### SYK inhibition reduces lesion size and parasite burden in cutaneous leishmaniasis

**Fig. 6.**
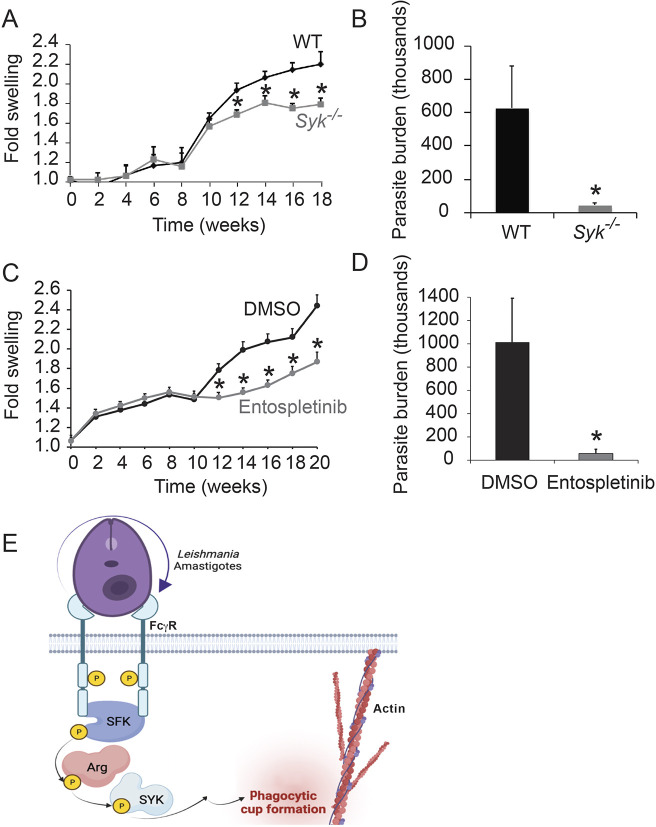
**SYK facilitates efficient infection in a mouse model of cutaneous leishmaniasis.** (A) Comparison of lesion size in WT versus *Syk^flox/flox^* LysM Cre+ mice. Ten mice per category were infected in the right hind foot with 1×10^6^ *L. amazonensis* metacyclic promastigotes and monitored over time. Three experiments were performed; shown is a representative experiment. Graphs show the mean+s.e. increase in infected foot width when compared to the uninfected foot. *P<0.05 by two-way ANOVA with Tukey's post hoc test, compared to WT mice. (B) *Syk^flox/flox^* LysM Cre+ mice have a lower parasite burden than WT mice. Plotted is the mean+s.e. number of parasites isolated from lesions at the termination of the experiment shown in A (week 18; *n*=10 mice per category). *P<0.05 by two-tailed paired *t*-test. (C) Comparison of lesion size in DMSO- and entospletinib-treated mice. Experiment performed as in A, except that mice were treated with 100 mg/kg/d of entospletinib in their drinking water or the equivalent volume of DMSO diluent, starting 4 d prior to infection and continuing throughout the experiment. *P<0.05 by two-way ANOVA with Tukey's post hoc test, compared to DMSO-treated mice. (D) Entospletinib-treated mice have a lower parasite burden than DMSO-treated mice. Shown is the mean+s.e. number of *L. amazonensis* parasites isolated from infected footpads at the termination of the experiment shown in C (week 20), calculated as in B. *P<0.05 by two-tailed paired *t*-test. (E) Relationship between FcγR signaling, SFKs, Arg and SYK during *Leishmania* amastigote uptake. Upon FcγR ligation by amastigotes, host cell Hck, Fgr and Lyn are activated. These SFKs phosphorylate and activate Arg kinase, which phosphorylates and activates SYK. SYK activates other kinases within the host cell (for example, CrkII), leading to actin polymerization and parasite uptake. Diagram created with BioRender (https://www.biorender.com).

Knowing that SYK was required for efficient uptake of *Leishmania* by Mφs, we investigated whether its activity was required for the manifestations of cutaneous leishmaniasis in mice. We infected *Syk^flox/flox^* LysM Cre+ mice and wild-type (WT) mice in the right hind foot with *L. amazonensis* promastigotes and monitored lesion size over time. We found that the *Syk^flox/flox^* LysM Cre+ mice developed smaller lesions than the WT mice (Fig. 6A), suggesting that SYK activity is necessary for maximal pathogenesis of cutaneous leishmaniasis in the mouse model.

The smaller lesions seen in *Syk^flox/flox^* LysM Cre+ mice potentially could be the result of differences in their immunological responses to infection. As a simplified paradigm, Th1 responses to leishmaniasis are typically protective, and Th2 responses are generally deleterious to the infected host (Jones et al., 1998). Therefore, we isolated draining lymph nodes from infected WT and *Syk^flox/flox^* LysM Cre+ mice and assayed cytokine secretion after stimulation with parasite lysates, comparing this to secretion after concanavalin A (Con A) stimulation (a positive control) or no stimulation (a negative control). Overall, we found limited differences between these two categories of mice (Table 1; Fig. S5A–E), with the exception of IL-1β induction, which SYK is known to facilitate (Callaway et al., 2015). However, decreases in IL-1β have been associated with worsening lesions (Lima-Junior et al., 2013). We also did not see any changes in the overall ratio of Th1 versus Th2 cytokine release (Table S1).

**Table 1.**
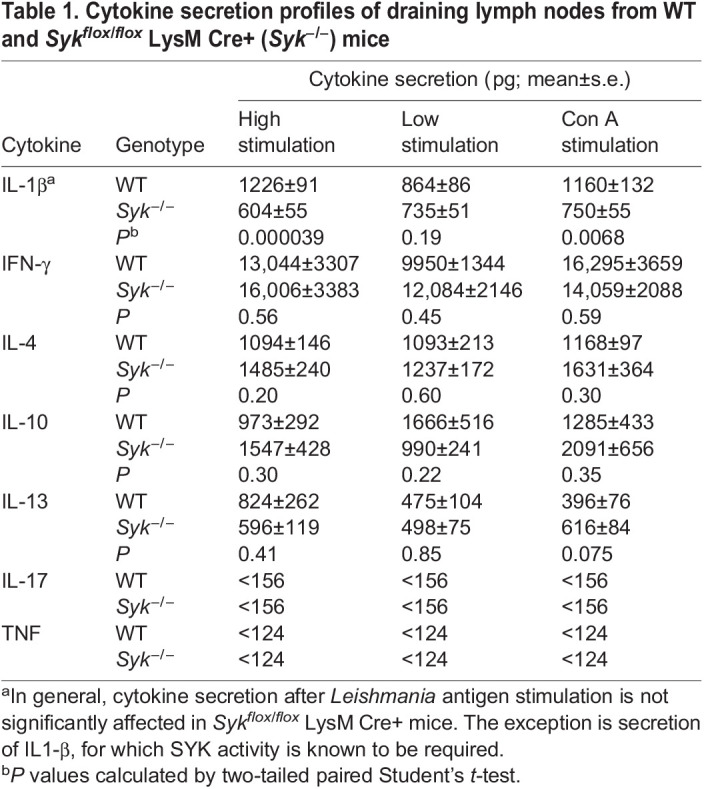
Cytokine secretion profiles of draining lymph nodes from WT and *Syk^flox/flox^* LysM Cre+ (*Syk^−/−^*) mice

Based on our data that SYK activity is required for *Leishmania* uptake by Mφs, we hypothesized that there would be fewer parasites contained within lesions of infected *Syk^flox/flox^* LysM Cre+ mice. Indeed, at experimental termination (week 18), we found that lesions in *Syk^flox/flox^* LysM Cre+ mice contained fewer parasites than those in WT mice (Fig. 6B).

Next, we tested whether inhibition of SYK using entospletinib decreased lesion size and parasite burden. We found that entospletinib-treated mice developed smaller lesions than DMSO-treated mice over time (Fig. 6C). There were minimal effects on cytokine secretion when draining lymph nodes isolated from DMSO-treated infected mice were treated with entospletinib during cell culture (Table 2; Fig. S5F–I). The lone statistically significant result was an increase in IL-13 in some stimulated entospletinib-treated samples. Finally, we demonstrated that lesions in entospletinib-treated mice contained significantly fewer parasites than those in DMSO-treated mice at the termination of the experiment (week 20; Fig. 6D).

**Table 2.**
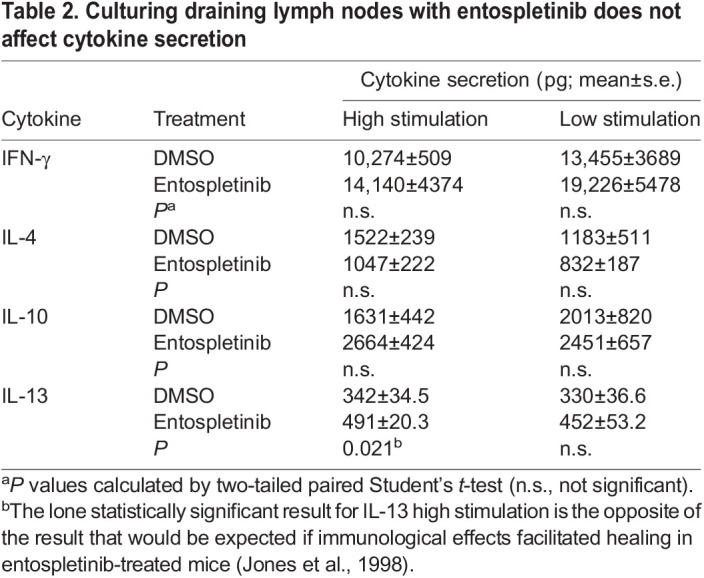
Culturing draining lymph nodes with entospletinib does not affect cytokine secretion

In summary, we have demonstrated that SFKs and Abl-family kinases activate SYK to facilitate phagocytosis, the uptake of *Leishmania* by Mφs and the disease manifestations seen in experimental cutaneous leishmaniasis. In combination with our previous results (Wetzel et al., 2012, 2016), these findings suggest that preventing parasite uptake by host cells using kinase inhibitors could be a valid strategy for designing novel antileishmanial therapies.

## DISCUSSION

We have shown previously that host SFKs and Abl-family kinases promote *Leishmania* uptake by Mφs (Wetzel et al., 2012, 2016). Our results here demonstrate that SYK also is necessary for maximal *Leishmania* promastigote and amastigote uptake by Mφs. Activation of SYK by SFKs and Abl-family kinases in Mφs facilitates *Leishmania* internalization. Finally, mice lacking SYK in monocytes or treated with the SYK kinase inhibitor entospletinib exhibit significantly reduced lesion severity and parasite burden when infected with *L. amazonensis*. Our data lead us to propose a relationship between FcγR signaling, SFKs, Arg and SYK during *Leishmania* amastigote uptake. Upon FcγR ligation by amastigotes, Hck, Fgr and Lyn are activated. These SFKs phosphorylate and activate Arg kinase, which phosphorylates and activates SYK, which leads to activation of CrkII, actin dynamics at the site of cell entry and formation/closure of the phagocytic cup (Fig. 6E).

Engagement of FcγR stimulates downstream responses important for host immunity and receptor-mediated phagocytosis. Our results demonstrate that FcγR-mediated signals permitting *Leishmania* amastigote uptake are relayed to SYK. SYK previously was shown to facilitate phagocytosis after binding of opsonized particles to FcγR on phagocytic cells (Crowley et al., 1997). Reductions in IgG-mediated phagocytosis by *Syk^−/−^* BMDMs were previously described to be secondary to defects in phagocytic cup formation and closure, rather than defects in Mφ activation (Crowley et al., 1997), which is supported by our data. The effects of the novel SYK inhibitor entospletinib on phagocytosis had not been explored to date. We find that fewer IgG-coated beads or *Leishmania* amastigotes are internalized by entospletinib-treated Mφs or Mφs deficient in SYK. Neither condition changes the numbers of beads or parasites bound to Mφs. The role of SYK in C3bi-mediated phagocytosis has been somewhat controversial (Kiefer et al., 1998; Tohyama and Yamamura, 2006; Walbaum et al., 2021). Our evidence supports SYK as a major contributor to efficient C3bi-mediated phagocytosis. Importantly, we also establish that SYK promotes the uptake of *Leishmania* promastigotes.

Multiple signaling pathways are regulated by SYK during phagocytosis, including linker for activation of T cells (LAT; Balagopalan et al., 2015), phospholipase Cγ (PLCγ; Karimi et al., 1999), and phosphoinositide 3-kinase (PI3-K; Yamamori et al., 2000). In addition, PI3-K signaling has been specifically implicated in *Leishmania* uptake by phagocytic cells (Cummings et al., 2012). In combination with previous studies, our experiments indicate that the specific signaling events necessary for FcγR-mediated *Leishmania* amastigote internalization are initiated by the activation of SFKs, which proceed to phosphorylate Arg kinase, which in turn phosphorylates SYK. Our data from experiments using entospletinib suggest that actin polymerization within phagocytic cups is increased, consistent with our previous findings using Mφs lacking Abl-family kinases (Wetzel et al., 2012). The pSYK antibody used here specifically recognizes phosphorylation on Tyr525 and Tyr526, which are SYK autophosphorylation sites that are necessary for maximal activity. Based on our data, we presume that Abl-family kinases are modulating the phosphorylation of these two residues during amastigote uptake. However, our data do not rule out the possibility that other sites on SYK are also phosphorylated. The link between Abl-family kinases and SYK is likely to be direct, as previous studies have demonstrated that purified Arg kinase phosphorylates SYK *in vitro* (Bonnerot et al., 1998; Greuber and Pendergast, 2012; Parsa et al., 2008). Similarly, SFKs have been shown to phosphorylate Arg kinase *in vitro* (Mader et al., 2011; Plattner et al., 2004; Tanis et al., 2003). We explored whether the extracellular domains of PSGL-1, which is known to activate SYK signaling via interactions with FcγR (Zarbock et al., 2008), also participate in this signaling cascade, but were unable to find evidence that this alternative receptor is relevant during *Leishmania* uptake. However, the methods we used would not address the possibility that signaling occurs through the intracellular domains of PSGL-1; additional experiments will be conducted for this purpose. In addition, this study, in combination with our prior work (Wetzel et al., 2012, 2016), demonstrates that activation of SFKs, Abl-family kinases and SYK after FcγR binding results in CrkII activation during the uptake of *Leishmania* parasites. A number of other downstream mediators of SYK signaling have been reported (Mócsai et al., 2010). Our future studies will directly test whether these mediators, or other kinases previously thought to facilitate phagocytosis or internalization of other pathogens, also participate in the uptake of *Leishmania*.

Because *Leishmania* parasites that remain extracellular within a mammalian host are thought to either die or be killed by the host immune system, we tested whether SYK deficiency would restrict disease manifestations in a mouse model of cutaneous leishmaniasis. Our studies indicate that *Leishmania* survival and pathogenesis in mice depends on SFKs (Wetzel et al., 2016), Abl and Arg (Wetzel et al., 2012, 2016), and now SYK. We believe that the smaller lesions seen in SYK-deficient mice primarily occur due to the deficits we identified in parasite internalization by phagocytes. Consistent with this hypothesis, there are fewer parasites within the lesions of SYK-deficient mice or mice treated with entospletinib. Since our hypothesis is difficult to test directly, our conclusions in part stem from eliminating other possibilities. For example, we found that entospletinib did not kill *Leishmania* parasites directly, and we saw no defects in parasite survival within entospletinib-treated Mφs.

Furthermore, we assessed whether the reductions in lesion size observed in SYK-deficient mice could be due to variations in the host immune response to leishmaniasis compared to that in control mice. For example, SYK deficiency is known to affect B- and T-cell development in mice and immune cell adhesion (Mócsai et al., 2010), which might adversely affect the ability of the host to control infections. However, our results suggest that such defects play a more limited role in our experimental system. The overall innate immune response to leishmaniasis is complex and dependent upon the infecting parasite species and the mouse strain used. In general, Th1 responses lead to lesion healing and Th2 responses result in lesion worsening (Jones et al., 1998). Our profiling of cytokines secreted by isolated lymph nodes after infection revealed no significant differences in Th1 versus Th2 responses between SYK-deficient and control mice. Lymph nodes from *Syk^flox/flox^* LysM Cre+ mice did exhibit defects in IL-1β secretion, which is known to be partly dependent on SYK (Callaway et al., 2015). However, we would expect that a decrease in IL-1β would worsen lesions based on prior studies (Lima-Junior et al., 2013). Similarly, we did not see differences in secretion of cytokines from lymph nodes isolated from infected mice that were incubated in entospletinib, save for an increase in IL-13 in some stimulated entospletinib-treated samples, which also should not mediate lesion healing (Jones et al., 1998). Therefore, we saw no differences in the immune response to leishmaniasis from our cytokine profiling that would explain the decreased lesion size in mice deficient in SYK or treated with entospletinib. However, further studies are needed to fully delineate changes in the immunological response to SYK inhibition in experimental murine leishmaniasis.

In summary, our studies demonstrate that SYK is required for maximal *Leishmania* uptake by Mφs and disease in mice. Our future work will continue to trace the signaling pathways necessary for internalization of this parasite by Mφs, with an eye towards using inhibitors of these pathways as treatment for leishmaniasis. A host-targeted therapeutic strategy is likely to be less susceptible to the development of resistance. Although our studies have thus far focused on *Leishmania*, our findings likely are more broadly applicable, as other intracellular pathogens also utilize and activate SYK to facilitate receptor-mediated uptake. For example, overexpression of membrane-targeted SYK in cells treated with Abl kinase inhibitors partially rescues the impairment in phagocytosis of *Francisella tularensis* (Parsa et al., 2008). Thus, there is potential to harness host kinases such as SYK as targets for a broad-spectrum antimicrobial agent against intracellular pathogens.

## MATERIALS AND METHODS

### Mice

The UT Southwestern Institutional Animal Care and Use Committee approved all animal protocols. *Syk^flox/flox^* LysM Cre+ and *Syk^flox/flox^* LysM Cre− littermates were generated by crossing LysM Cre+ mice to *Syk^flox/+^* mice (all on a C57BL/6J background, which along with WT C57BL/6J mice had been purchased from Jackson Labs, Bar Harbor, ME, USA). *Hck^−/−^ Fgr^−/−^ Lyn^−/−^*, *Arg^−/−^* and *Abl^flox/flox^* LysM Cre+ mice were generated as described previously (Wetzel et al., 2012). For all experiments, WT littermates were paired with the kinase mutant under study to limit any effects from variation in genetic backgrounds.

### Mammalian cell culture

RAW 264.7 cells (obtained from ATCC, Manassas, VA, USA) were incubated in Dulbecco's modified Eagle's medium (DMEM; Thermo Fisher Scientific, Waltham, MA, USA) plus 10% heat-inactivated fetal bovine serum (FBS; Gemini) and 10 µg/ml penicillin/gentamicin (Sigma). Bone marrow-derived primary Mφs (BMDMs) were harvested from tibias of *Syk^flox/flox^* LysM Cre+*, Syk^flox/flox^* LysM Cre−*, Hck^−/−^ Fgr^−/−^ Lyn^−/−^,* WT, *Arg^−/−^* and/or *Abl^flox/flox^* LysM Cre+ mice, and confirmed as described previously (Wetzel et al., 2012). All cells were screened for *Mycoplasma* contamination (Wetzel et al., 2016).

### *Leishmania* culture

*L. amazonensis* strain IFLA/BR/67/PH8 promastigotes (obtained from Norma W. Andrews, University of Maryland, College Park, MD, USA) were grown in Schneider's *Drosophila* medium (Sigma, St. Louis, MO, USA) with 15% heat-inactivated FBS and 10 µg/ml penicillin/gentamicin (Sigma) (Wetzel et al., 2012). 7-day-old cultures were used for uptake experiments to maximize yield of infective metacyclic promastigotes. *L. amazonensis* strain IFLA/BR/67/PH8 amastigotes were grown axenically (outside of mammalian cells) in supplemented Schneider's medium at pH 5.0, as described previously (Hodgkinson et al., 1996). All parasites were serially passaged through mice so that virulence would be maintained.

### Transgenic *Leishmania* strains

For intracellular survival assays, *L. amazonensis* expressing a bright monomeric green fluorescent protein, mNeonGreen, was used. mNeonGreen was cloned into the pLEXSY.hyg2 (Jena Bioscience, Jena, Germany) expression vector with a hygromycin resistance marker, as previously described (Ullah et al., 2020). pLEXSY.hyg2 containing the mNeonGreen gene (pLEXSY.hyg2-mNeonGreen) was transfected into *L. amazonensis* promastigotes using the Human T-Cell Nucleofector kit and the Amaxa Nucleofector electroporator (program U-033; Lonza, Basel, Switzerland), for integration into the 18S rRNA locus within the nuclear DNA. Following transfections, promastigotes were allowed to grow for 24 h at 26°C and then selected with 100 μg/ml hygromycin in Schneider's *Drosophila* medium. Clones were isolated via limiting dilution, and the cultures were subsequently maintained in Schneider's *Drosophila* medium supplemented with 100 μg/ml hygromycin. To maintain virulence, mNeonGreen-expressing *L. amazonensis* parasites were passaged regularly in C57BL/6 mice, as described previously (Wetzel et al., 2016).

### Phagocytosis assays

BMDMs and RAW 264.7 cells were plated at >50% confluence and starved overnight in macrophage-colony stimulating factor (M-CSF)-starved or serum-free media (as indicated in the ‘Mammalian cell culture’ section). For drug experiments, except where indicated, compounds were obtained from LC Laboratories (Woburn, MA, USA). Mφs were incubated in 3.3 µM imatinib, 1 µM SU6656, 1 µM entospletinib or 0.1% DMSO (Sigma) for 2 h. For C3bi-coated bead internalization only, Mφs were also pre-activated with phorbol 12-myristate 13-acetate (PMA; Sigma) for 1 h. As described previously (Wetzel et al., 2012, 2016), 2 µm latex green beads (Sigma) were incubated with human IgM (I-8260, Sigma), then in rabbit anti-human IgM (Sigma, 270A; for IgG-coated beads) or fresh mouse serum (for C3bi-coated beads). Ten beads/Mφ were added to Mφs for 30 min at 37°C. To distinguish internal from external beads, samples were fixed in 3% formaldehyde for 15 min, blocked in 2% BSA (no permeabilization; Sigma) (Wetzel et al., 2004, 2003, 2005), incubated in rabbit anti-human IgM (1:1000) and Hoechst 33258 (Sigma), and then incubated in Alexa-Fluor-564-conjugated goat anti-rabbit IgG (1:250; Thermo Fisher Scientific). Samples were visualized using a Cytation 5 imager (BioTek, Santa Clara, CA, USA) and analyzed by an observer who was unaware of the experimental conditions. Representative images for beads were collected on the Cytation 5 using a 40× objective and processed linearly in Adobe Photoshop (version 13.0.6) (Ullah et al., 2020). The phagocytic index (PI) was calculated as the number of beads internalized per 100 Mφs, and experimental samples were normalized to controls. The total number of beads per 100 Mφs was also calculated, with experimental samples were normalized to controls. At least 100 Mφs and 100 beads were counted from multiple imaged fields per condition. All experiments had at least three biological replicates performed, and means were calculated. Each biological replicate was then normalized so that the control was set to 100% for each experiment. The experimental category shown represents a percentage of this control value with s.e. A two-tailed one-sample *t-*test was used for statistical analysis of these results (Wetzel et al., 2012, 2016).

### *Leishmania* uptake assays

RAW 264.7 cells or BMDMs were treated with DMSO, PMA and inhibitors as described above. To allow C3bi opsonization, we incubated metacyclic promastigotes in murine serum for 1 h (as in Wetzel et al., 2012). To allow IgG opsonization, we incubated amastigotes with anti-P8 primary IgG1 antibody (Pan and McMahon-Pratt, 1988) for 1 h. Opsonized parasites were incubated with Mφs at a 10:1 promastigotes to Mφ ratio or a 2:1 amastigotes to Mφ ratio (Wetzel et al., 2012, 2016). Samples were fixed with 3% formaldehyde, blocked in 2% BSA in PBS, incubated in mouse anti-gp46 antibody (promastigotes; 1:50) or mouse anti-P8 antibody (1:1000) (both supplied by Diane McMahon-Pratt, Yale University, CT, USA; Hodgkinson et al., 1996), then incubated in Alexa-Fluor-568 goat anti-mouse secondary antibody (1:250; A10037, Thermo Fisher Scientific) (Wetzel et al., 2012). After permeabilization, parasites were incubated in their original primary antibody, then in Alexa-Fluor-488 donkey anti-mouse secondary antibody (1:250; A21202, Thermo Fisher Scientific) and Hoechst 33258 (Sigma). Where applicable, far-red phalloidin staining (A22287, Thermo Fisher Scientific) was employed at 1:100. Visualization, analysis, and statistics were performed as described above for bead phagocytic assays (Wetzel et al., 2012, 2016). Representative images for parasites were collected on a Zeiss LSM 880 inverted confocal Airyscan microscope using a 40× objective (Ullah et al., 2020). Shown are maximal intensity projections constructed from full *Z*-thickness stacks through parasites and Mφs using Image J (1.52a, http://imagej.nih.gov/ij) and processed linearly in Adobe Photoshop (version 13.0.6) (Ullah et al., 2020).

For PSGL-1 experiments, amastigotes were incubated with anti-P8 primary IgG1 antibody (Pan and McMahon-Pratt, 1988) for 1 h. RAW 264.7 cells were pre-treated with anti-mouse CD162 (PSGL-1) monoclonal antibody (4RA10; 12-1621-80, Thermo Fisher Scientific), entospletinib, both, or equal amounts of vehicle (DMSO) for 1 h. The anti-mouse CD162 antibody was employed at 1:2500, 1:1000, 1:500, 1:250 and 1:100, and entospletinib was applied at 1 μM. Opsonized parasites were incubated with RAW 264.7 cells at a 10:1 parasite to RAW 264.7 cell ratio. Samples were fixed with 4% formaldehyde, blocked in PBS containing 5% BSA, incubated in mouse anti-P8 antibody (1:1000), then incubated in Alexa-Fluor-568 goat anti-mouse secondary antibody (1:250; A10037, Thermo Fisher Scientific) (Wetzel et al., 2012). After permeabilization with 0.1% Triton X-114, parasites were incubated in their original primary antibody, then in Alexa-Fluor-488 donkey anti-mouse secondary antibody (1:250; A21202, Thermo Fisher Scientific) and Hoechst 33258 (Sigma). Visualization was performed on an INCell Analyzer 6000 (GE Healthcare) microscope using a 40× objective in the UT Southwestern High Throughput Screening Core facility. For Fig. S3C, images were processed through a CellProfiler pipeline (https://cellprofiler.org). Three to six technical replicates were performed for each condition, and three separate biological replicates were combined to provide the data shown. Statistics were performed as above for phagocytic assays (Wetzel et al., 2012, 2016). Fig. S3D was analyzed as indicated in the ‘Phagocytosis assays’ and ‘*Leishmania* uptake assays’ sections of the Materials and Methods.

### Viability assays

*L. amazonensis* promastigotes, axenic amastigotes and RAW 264.7 cells were cultured as above, added to 96-well plates containing dilution series of relevant compounds and incubated for 72 h before measuring viability using alamarBlue (10%; Thermo Fisher Scientific; Ullah et al., 2020). Plates were read at 6 h using a BioTek Synergy H1 plate reader (530 nm excitation, 570 nm emission; Ullah et al., 2020). To assess parasite survival, ten *L. amazonensis* promastigotes per Mφ were added to G-CSF-starved WT BMDMs and incubated for 4 h. After five washes with PBS, DMSO or 1 µM entospletinib was added in medium supplemented with 10% supernatant from L929 cell culture (L929 cells were grown in the laboratory and obtained from ATCC). Samples were analyzed after 72 h by microscopy using the Cytation 5 imager, and total numbers of amastigotes/Mφ were calculated by an observer unaware of the experimental conditions, using the methods described above for phagocytosis assays.

### Immunoblotting

To measure phosphorylation of SYK and CrkII, RAW 264.7 cells were incubated overnight in serum-free medium, with experiments performed at ∼70% confluence. To initiate experiments, RAW 264.7 cells were pre-incubated in 3.3 µM SU6656, 3.3 µM imatinib, 1 µM entospletinib, 50 nM DPH or DMSO for 2 h. IgG-opsonized amastigotes were added to adherent starved RAW 264.7 cells (at a ratio of 20:1) for 30 min (Pan and McMahon-Pratt, 1988). Cells were lysed using Pierce radioimmunoprecipitation (RIPA) buffer (89901, Thermo Fisher Scientific, Rockford, IL, USA) with protease and phosphatase inhibitors (1:10,000, Cocktail 1, Thermo Fisher Scientific), and protein concentrations were assessed using bicinchoninic acid (BCA) (23225, Thermo Fisher Scientific). For representative images, equivalent protein amounts were loaded on 10% SDS–PAGE gels, transferred to nitrocellulose membranes, and probed with antibodies against phosphorylated Syk (pSyk; Tyr525/526; 2710S; Cell Signaling Technology, Danvers, MA, USA) at 1:500, Syk (2712S, Cell Signaling Technology) at 1:500, phosphorylated CrkII (pCrk; 3491, Cell Signaling Technology) at 1:500, or CrkII (CRK; MAS-15891, Invitrogen) at 1:500. Immunoblotting with rabbit anti-GAPDH (2118L, Cell Signaling Technology) at 1:1000 was used as a loading control. HRP-conjugated goat anti-mouse and anti-rabbit IgGs (7076 and 7074, Cell Signaling Technology) at 1:2000 were used as secondary antibodies. For analysis, relative amounts of pSyk or pCrk were compared with ImageJ analysis software and were normalized to Syk or Crk (membranes were stripped of pSyk or pCrk and reprobed for Syk or Crk). Imaging was performed by using a phosphorimager (ImageQuant LAS 4000, GE, Boston, MA, USA). Each experiment was performed four times. Two-way ANOVA with Tukey's multiple comparison test was used to determine statistical significance.

### Murine infections

8-week-old female C57BL/6 mice were infected in the dorsal side of the right hind foot with 1×10^6^ metacyclic promastigotes (isolated via Percoll gradient, Sigma; Wetzel et al., 2012) per mouse; ten mice were used per experimental condition. All animals surviving until termination were included in analysis. For entospletinib-treated mice, three experiments were conducted. Mice were provided 100 mg/kg/d of entospletinib or the diluent (0.1% DMSO) in their drinking water at pH 5.5, starting 4 d prior to the experiment and continuing through to termination (Wetzel et al., 2016). For *Syk^flox/flox^* LysM Cre+ mice, three independent experiments were conducted. Lesion size was measured every other week with calipers by an investigator unaware of the experimental conditions, and infected:uninfected foot thickness ratios were calculated and compared to time of infection using a two-way ANOVA. The number of parasites in lesions were determined at the time of experiment termination (between 18 and 20 weeks post infection) (Soong et al., 1997).

Lymph nodes from infected mice were harvested for cytokine profiling as described previously (Wetzel et al., 2012, 2016). In brief, cells from lymph nodes (5×10^6^ cells) were plated and stimulated with varying concentrations of promastigote lysates (high stimulation, 2.5×10^6^ parasites; low stimulation, 5×10^5^ parasites) or concanavalin A (Con A) (5 µg/ml, Sigma); supernatants were harvested at 72 h. An unstimulated negative control (medium alone) was employed to ensure that cytokines released were secondary to stimulation (data not shown), and levels of all measured cytokines were found to be negligible compared to background. Levels of IL-1β, IL-4, IFN-γ, IL-10, IL-13 and IL-17 were assessed by ELISA (as in Wetzel et al., 2016) and compared to background (unstimulated) levels. To assay the effects of entospletinib on cytokine secretion, isolated draining lymph nodes from four infected DMSO-treated WT mice were cultured as described above, with either high or low stimulation, except that DMSO or 2 µM entospletinib was added to the cells from each of these four isolated draining lymph nodes during culture for 72 h. Cytokine ELISAs were performed on harvested supernatants as described above.

## Supplementary Material

10.1242/joces.260809_sup1 Supplementary information
