# Supplementary material for: Src- and Abl-family kinases activate spleen tyrosine kinase to maximize phagocytosis and Leishmania infection
Source: J Cell Sci. 2023 Jul 28;136(14):jcs260809. doi: 10.1242/jcs.260809 (PMC10399977; doi:10.1242/jcs.260809)
Supplement: Supplementary information [file joces-136-260809-s1.pdf]

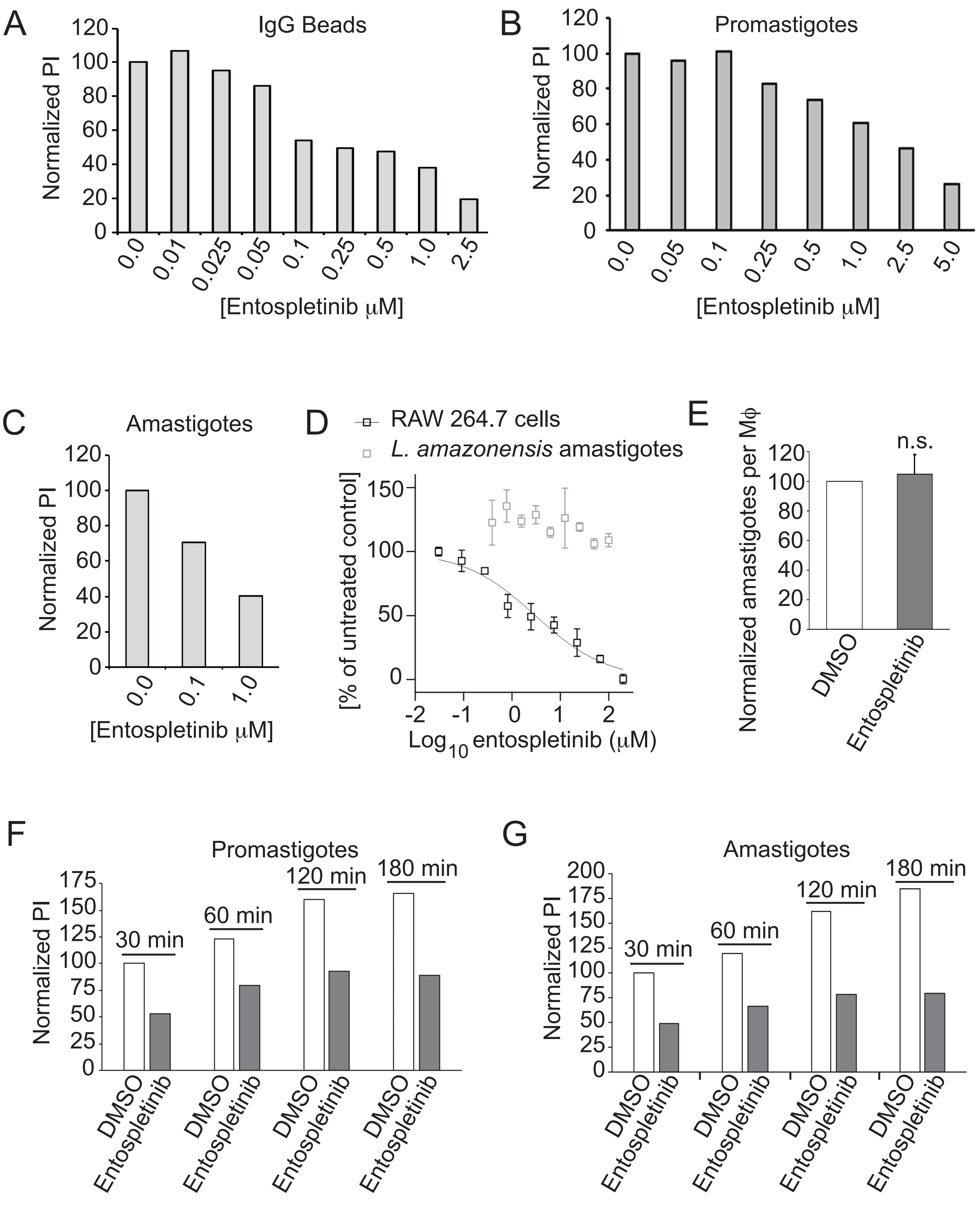

Journal of Cell Science • Supplementary information

Fig. S1. Titrations of entospletinib dosage and effects over time. (A) Titration of entospletinib's effects on IgG-coated bead uptake by RAW 264.7 cells. Mϕs were treated with DMSO (shown as 0.0 μM entospletinib) or increasing concentrations of entospletinib for 2 h and incubated with IgG-coated beads for 30 m. Shown is one representative experiment of 2 experiments. Data normalized to DMSO category (PI = 100%). (B) Titration of entospletinib's effects on promastigote uptake by RAW 264.7 cells. Mϕs were treated with increasing concentrations of entospletinib or DMSO for 2 h and incubated with IgG-coated amastigotes for 30 m. Shown is one representative experiment of 2 experiments. (C) Titration of entospletinib's effects on amastigote uptake by RAW 264.7 cells. Mϕs were treated with DMSO, 0.1 μM, or 1 μM entospletinib and incubated with IgG-coated amastigotes for 30 m. Shown is one representative experiment of 2 experiments. (D) Entospletinib log concentration-response curves for RAW 264.7 cells (black) and axenic *L. amazonensis* amastigotes (grey) for one representative experiment of 3 biological replicate experiments. Triplicate technical replicates were used. Cells were incubated for 72 h in the concentrations of entospletinib shown. (E) Treatment with 0.5 μM entospletinib has no effects on *L. amazonensis* amastigote survival inside RAW 264.7 cells. Mϕs were allowed to internalize *L. amazonensis* amastigotes expressing mNeonGreen, and then, after 24 h, they were incubated with DMSO or 0.5 μM entospletinib for 72 h. Shown is the mean number of *L. amazonensis* amastigotes per 100 entospletinib-treated Mϕ, normalized to DMSO-treated Mϕ (100%), ± SE. n = 3 separate biological replicates. (F) The relative decrease in promastigote PI in entospletinib-treated RAW 264.7 cells does not change after long drug incubations. Mϕs were treated with 1 μM entospletinib or DMSO for 2 h and incubated with C3bi-coated promastigotes for 30, 60, 120, or 180 m. Data normalized to DMSO, 30 m category. Shown is one representative experiment of 2 experiments. (G) The relative decrease in amastigote PI in entospletinib-treated RAW 264.7 cells does not change after long drug incubations. Experiment performed as in (F), except that Mfs were incubated with IgG-opsonized amastigotes. Shown is one representative experiment of 2 experiments.

Journal of Cell Science • Supplementary information

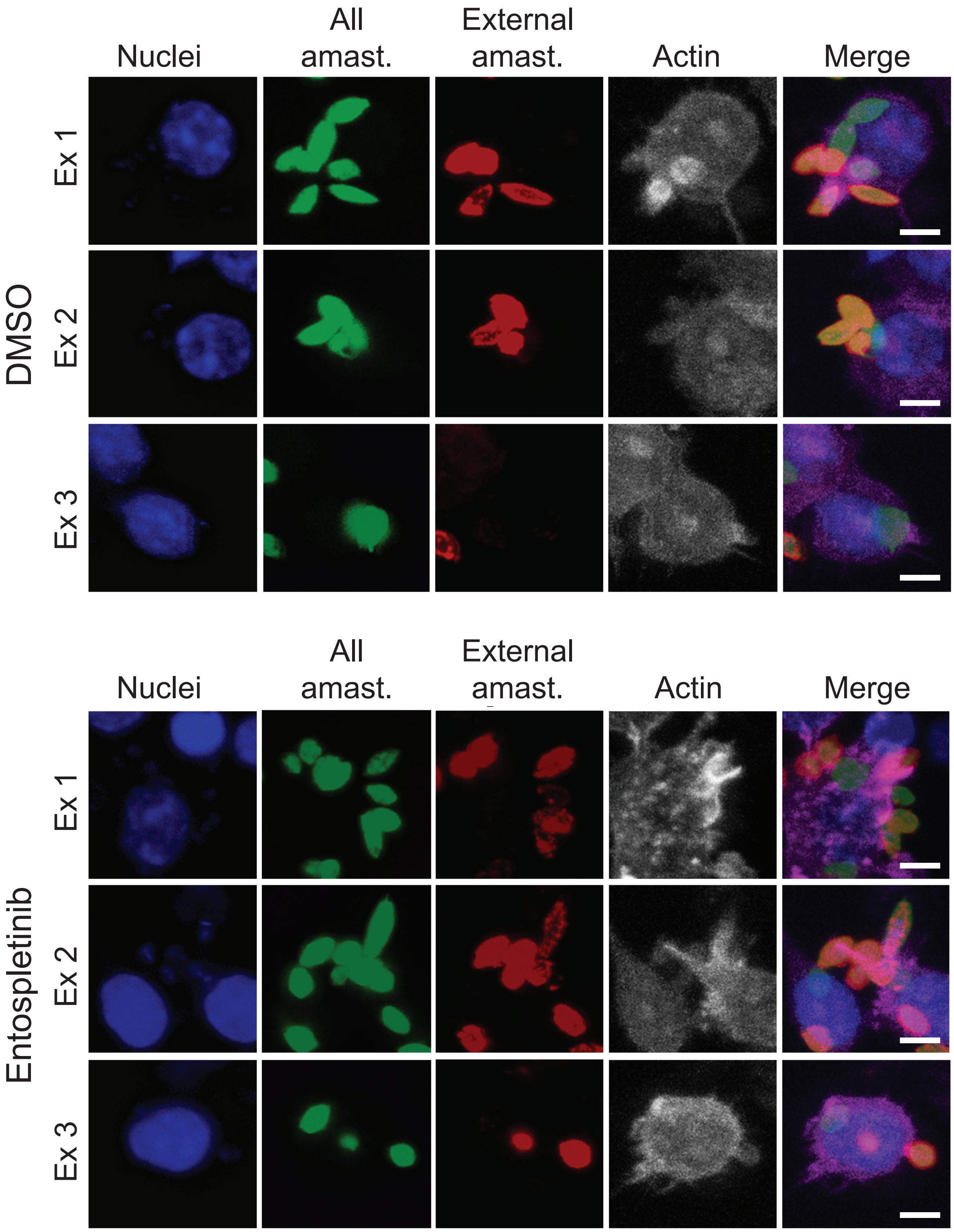
Nuclei
All amast.
External amast.
Actin
Merge
DMSO
Ex 1
Ex 2
Ex 3
Nuclei
All amast.
External amast.
Actin
Merge
Entospletinib
Ex 1
Ex 2
Ex 3

Fig. S2. Representative phagocytic cups in DMSO vs entospletinib-treated Mϕs taking up *Leishmania* amastigotes. Shown are three separate examples of phagocytic cups in DMSO versus entospletinib-treated RAW 264.7 cells that are in the process of internalizing amastigotes. From left to right: Mϕ nuclei (blue, Hoescht); all amastigotes (green; p8); external amastigotes (red); actin (white/pink; phalloidin). Scale bar = 2 μm. Circular phagocytic cups like those seen in example 1 in the DMSO-treated Mϕs were not seen in entospletinib-treated Mϕs, consistent with prior literature suggesting that there are defects in cup closure in *SYK*$^{-/-}$ Mϕs (Crowley *et al.*, 1997). Actin staining also appears brighter in entospletinib-treated Mϕs than DMSO-treated Mϕs (quantified in Fig. S3B).

Journal of Cell Science • Supplementary information

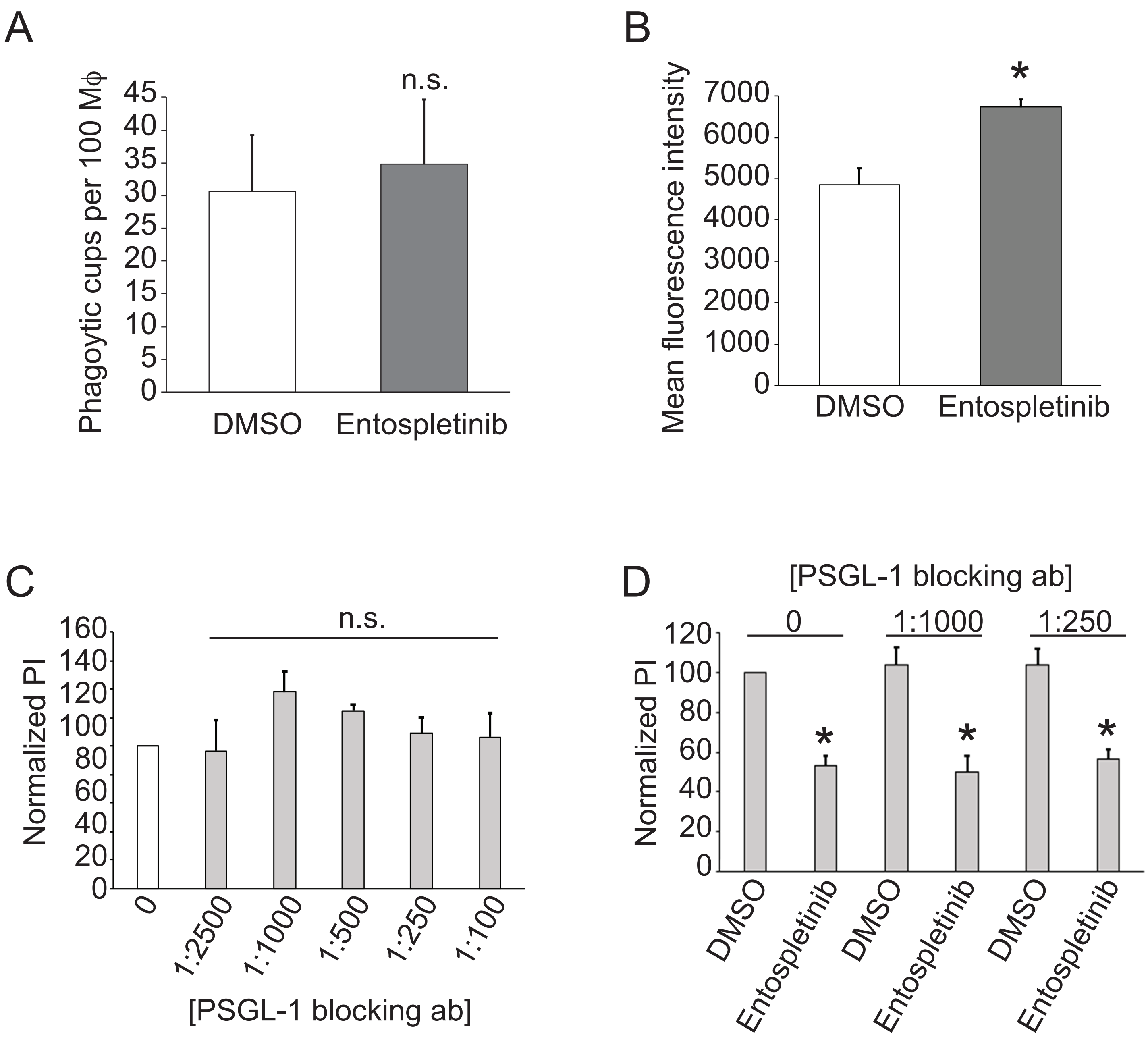

Journal of Cell Science • Supplementary information

Fig. S3. Mechanisms of SYK signaling during *Leishmania* uptake. (A) Despite differences in PI, the number of phagocytic cups seen between entospletinib-treated and DMSO-treated RAW 264.7 cells incubated with amastigotes is the same. This result is consistent with prior literature documenting defects in phagocytic cup closure (not formation) in *Syk*$^{-/-}$ phagocytes. (B) Actin in phagocytic cups shown in Fig. S2 is brighter in entospletinib-treated Mϕs than in DMSO-treated Mϕs. Relative fluorescence intensity was quantified in ImageJ by an observer blinded to experimental category. Shown is the mean intensity per field among at least 5 fields per category ± SE. *, $p < 0.05$ by *t*-test. (C) Incubation with 4RA10, an antibody to P-selectin glycoprotein ligand 1 (PSGL-1), does not affect uptake of *Leishmania* amastigotes by Mϕs. Mϕs were treated with increasing concentrations of 4RA10 for 2 h and incubated with IgG-coated amastigotes for 30 m to allow internalization as described above. Shown is the mean PI ± SE from 3 biological replicates. All categories non-significant by ANOVA. (D) The entospletinib-induced reduction in amastigote PI is not affected by 4RA10. Mϕs were treated with increasing concentrations of 4RA10 and DMSO vs 1 μM entospletinib for 2 h, then incubated with IgG-coated amastigotes for 30 m to allow internalization as described above. Shown is the mean PI ± SE from 3 biological replicates. *, $p < 0.05$ by ANOVA.

Journal of Cell Science • Supplementary information

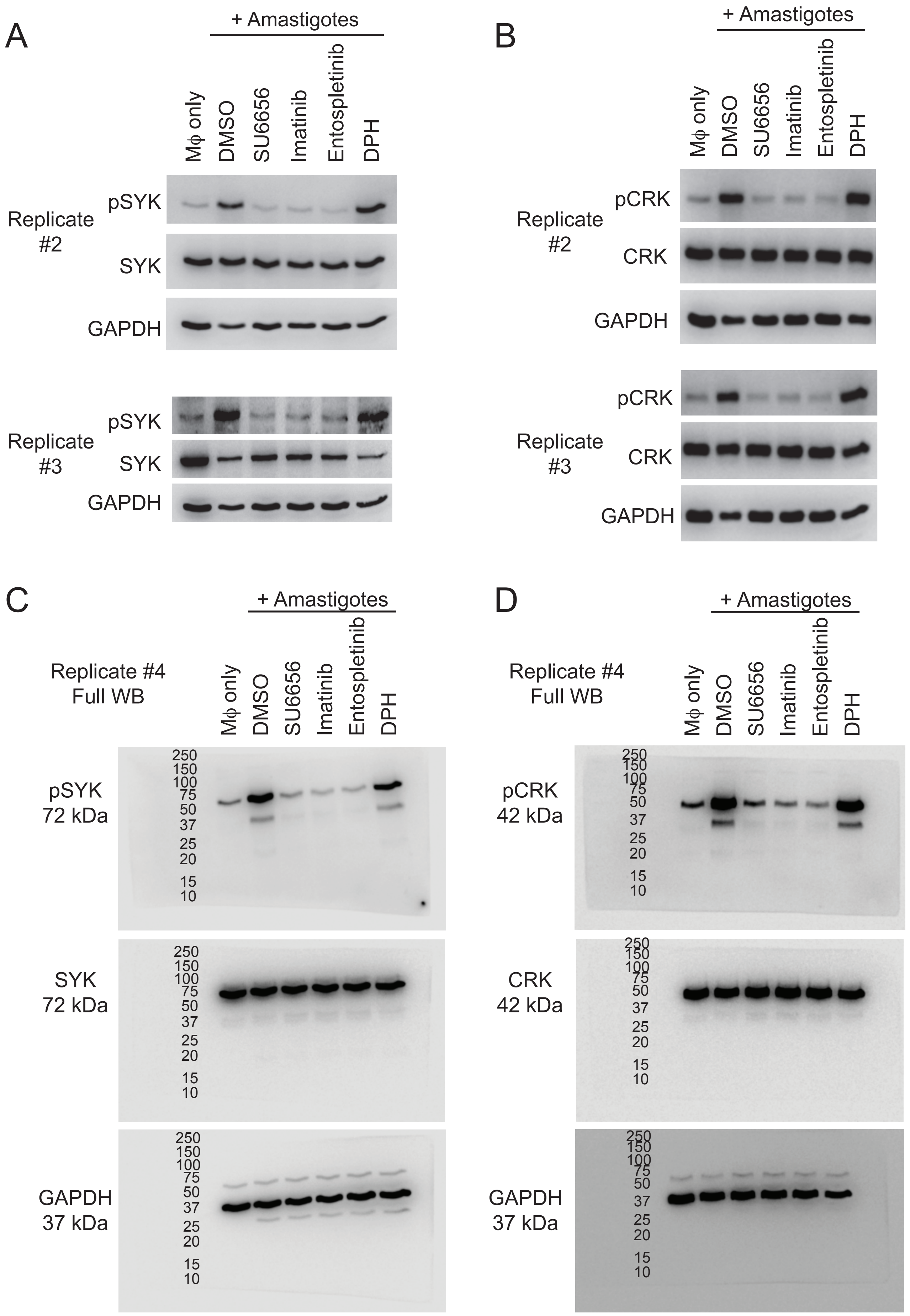

Journal of Cell Science • Supplementary information

Fig. S4. Western Blots. All Western blots quantified in Figure 5 are shown. (A) Biological replicates 2 and 3 of Western blots quantified for pSYK calculations. (B) Biological replicates 2 and 3 of Western blots quantified for pCRK calculations. (C) Full Western blot for biological replicate 4 used for pSYK calculations. (D) Full Western blot for biological replicate 4 used for pCRK calculations.

Journal of Cell Science • Supplementary information

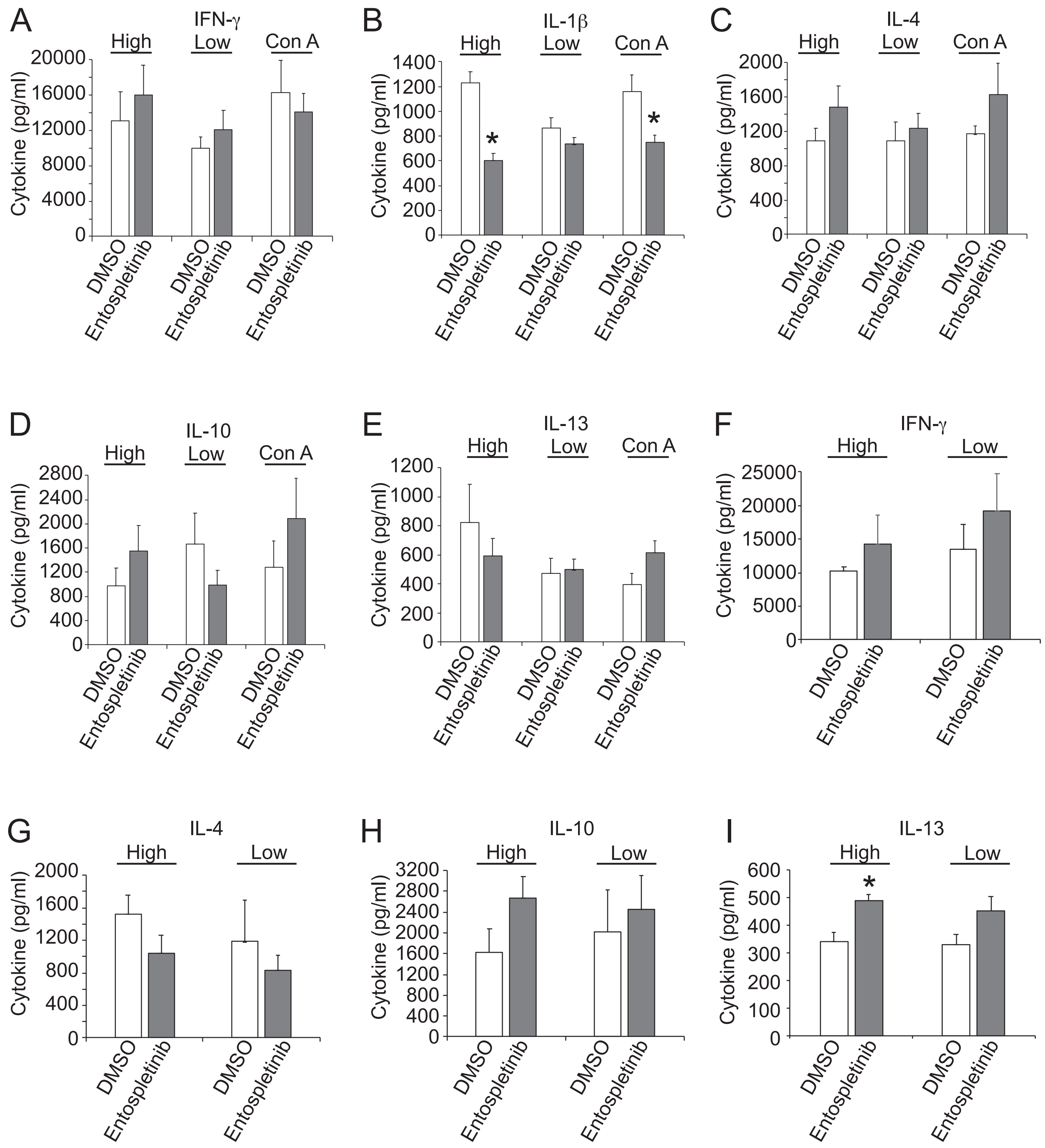

Fig. S5. Graphical representations of cytokine secretion data. (A-E) Graphs of data shown in Table 1, separated out by cytokine. A = IFN-γ, B= IL-1β, C = IL-4, D = IL-10, E = IL-13. (F-I) Graphs of data shown in Table 2, separated out by cytokine. F = IFN-γ, G = IL-4, H = IL-10, I = IL-13. *, $p < 0.05$ by *t*-test.

Journal of Cell Science • Supplementary information

Table S1. Cytokine secretion is not skewed towards a Th1 or Th2 response during *L. amazonensis* infection of *Syk*$^{flox/flox}$ LysM Cre+ mice compared to WT mice. Ratios of IFNγ:IL-4, IFNγ:IL-10, IFNγ:IL-13, and IFNγ:IL-4+10+13 were calculated from the data shown in Table 1.

Ratio	WT – High stimulation	*Syk*$^{-/-}$ – High stimulation	WT – Low stimulation	*Syk*$^{-/-}$ – Low stimulation
IFNγ:IL-4	11.9	10.8	9.1	9.8
IFNγ:IL-10	13.4	10.4	6.0	12.2
IFNγ:IL-13	21.9	27.0	20.9	24.2
IFNγ: IL-4+10+13	4.9	4.4	3.1	4.4

Journal of Cell Science • Supplementary information
