# Supplementary material for: Src- and Abl-family kinases activate spleen tyrosine kinase to maximize phagocytosis and Leishmania infection
Source: J Cell Sci. 2023 Jul 28;136(14):jcs260809. doi: 10.1242/jcs.260809 (PMC10399977; doi:10.1242/jcs.260809)
Supplement: Supplementary file 1 [file joces-136-260809_review_history.pdf]

Src- and Abl-family kinases activate spleen tyrosine kinase to maximize phagocytosis and *Leishmania* infection

Imran Ullah, Umaru Barrie, Rebecca M. Kernen, Emily T. Mamula, Francis Tho Huu Khuong, Laela M. Booshehri, Emma L. Rhodes, James M. Bradford, Arani Datta and Dawn M. Wetzel
DOI: 10.1242/jcs.260809

Editor: Daniel Billadeau

Review timeline

Original submission:	17 November 2022
Editorial decision:	16 January 2023
First revision received:	2 June 2023
Accepted:	19 June 2023

Original submission

First decision letter

MS ID#: JOCES/2022/260809

MS TITLE: The Src and Abl family kinases activate the Spleen Tyrosine Kinase to maximize phagocytosis and Leishmania infection

AUTHORS: Imran Ullah, Umaru Barrie, Rebecca M. Kernen, Emily T. Mamula, Arani Datta, Laela M. Booshehri, Emma L. Rhodes, James M. Bradford, and Dawn M. Wetzel
ARTICLE TYPE: Research Article

We have now reached a decision on the above manuscript.

To see the reviewers' reports and a copy of this decision letter, please go to: https://submit-jcs.biologists.org and click on the 'Manuscripts with Decisions' queue in the Author Area. (Corresponding author only has access to reviews.)

As you will see, the reviewers raise a number of criticisms that prevent me from accepting the paper at this stage. They suggest, however, that a revised version might prove acceptable, if you can address their concerns. If you think that you can deal satisfactorily with the criticisms on revision, I would be pleased to see a revised manuscript. We would then return it to the reviewers.

Please ensure that you clearly highlight all changes made in the revised manuscript. Please avoid using 'Tracked changes' in Word files as these are lost in PDF conversion.

I should be grateful if you would also provide a point-by-point response detailing how you have dealt with the points raised by the reviewers in the 'Response to Reviewers' box. Please attend to all of the reviewers' comments. If you do not agree with any of their criticisms or suggestions please explain clearly why this is so.

© 2023. Published by The Company of Biologists under the terms of the Creative Commons Attribution License (https://creativecommons.org/licenses/by/4.0/).

Reviewer 1

Advance summary and potential significance to field

I have read “The Src and Abl family kinases activate the Spleen Tyrosine Kinase to maximize phagocytosis and Leishmania infection” by Ullah et al. This paper describes a carefully conducted study illustrating the importance of Syk kinase of IgG-coated Leishmania amastigotes by macrophages. While not particularly surprising, the study is exceedingly well conducted using both knockout macrophages and inhibitors to delineate these effects. Moreover, it makes clear that an inhibitor of Syk under clinical investigation may be of some utility here. In addition, this work contributes to the understanding that Syk contributes to complement-mediated phagocytosis and the less well-understood concept that Abl1/2 contributes to both. Overall I believe that this paper will be of interest to the readership of JCS. I have a few minor comments that may (or may not) be of use to authors.

Comments for the author

Minor comments:
Fig1a. Why focus on a region where there are almost no beads in the middle of the FOV?
Fig 6B It might help to note in line 754 that the day the parasite burden was endpoint (day X).
Tables 1 and 2 are relatively difficult to interpret without an unstimulated control. E.g. it is hard to know what ‘low’ means relative to ‘high’.
I am not getting much out of the cytokine data in the discussion – mostly because it is unclear what the homeostatic state was for these measures. I think it is fine to leave the data in, but maybe just note that more work would be needed to delineate differences?
Line 319: “Our data add to the evidence that SYK is necessary for efficient C3bi-mediated phagocytosis.”
The density of compliment or IgG can make a huge difference – e.g. even RBCs will be taken up by Syk-/- macrophages if enough IgG is put on them. Maybe another way to say this is that ‘our evidence supports Syk as a major contributor to efficient…”

Reviewer 2

Advance summary and potential significance to field
In this study the authors have intended to demonstrate that Spleen Tyrosine Kinase(SYK) which is activated by Src and Abl protein kinases, increases the phagocytic activity and Leishmania infection. Both C3bi and IgG-mediated phagocytosis and internalization of promastigotes and amastigotes is facilitated by SYK. So, if treatment is provided against SYK it would be beneficial for Leishmaniasis.

The study is predictable, as the authors themselves mentioned that “SFK inhibitors and Abl family kinase inhibitors decrease the uptake of Leishmania parasites (Wetzel et al., 2012; Wetzel et al., 2016). And Others have suggested that signaling during phagocytosis by these two kinase families can be relayed to SYK (Crowley et al., 1997; Greuber and Pendergast, 2012). The study is merely correlative and did not explore pathways regulated by SYK in phagocytosis, such as LAT (linker for activation of T cells), phosphatidylinositol 3-kinase (PI 3-K), and phospholipase Cγ (PLCγ). Neither they have shown which step of phagocytosis is specifically inhibited, as phagocytosis is a multi-step process. I had failed to discriminate between internalization and phagocytosis from their results. The other few concerns have been listed below.

1.SYK can be activated through different pathways .In some research articles it has been also demonstrated that adhesion molecule P-selectin glycoprotein ligand 1(PSGL1) found in cells associates with SYK and activates it. Experiments need to be performed in order to check the activity of PSGL1 in case of Leishmania infection. Valid explanation needs to be provided that why only Src-Abl-SYK pathway is being focused in this hypothesis.
2. As all the experiments need to performed atleast 3 times, error bar needs to be provided to all the graphical representation.
3. Fig 2A needs to be explained clearly.

© 2023. Published by The Company of Biologists under the terms of the Creative Commons Attribution License (https://creativecommons.org/licenses/by/4.0/).

4.Quantification needs to be provided properly for Fig1A and Fig2A.
5.As uptake of both promastigotes and amastigotes are shown, effect of concentration of entospletinib in promastigote uptake in RAW 264.7 also needs to be shown.
6. Effect of time in promastigote uptake while incubating with entospletinib needs to be shown.
7. Quantification of Fig 3A needs to be provided.
8. In Fig 5A the last lane of the blots are deformed compared to others. Replace the blot through fresh experiment.
9. In Fig 5 pSYK and pCrk is only seen in presence of amastigotes and not promastigotes. Clear explanation needs to be provided 11. A valid explanation is required why different cell lines are used.
12. The data for cytokine secretion could be represented graphically.
13. Grammatical error in line no 169 and 268.
14. Better schematic diagram could be provided for the hypothesis.
15. Line 183 states that Fig 2A and 2B represent the uptake of promastigote and amastigote which do not match with figure legend.

Comments for the author

In this study the authors have intended to demonstrate that Spleen Tyrosine Kinase(SYK) which is activated by Src and Abl protein kinases, increases the phagocytic activity and Leishmania infection. Both C3bi and IgG-mediated phagocytosis and internalization of promastigotes and amastigotes is facilitated by SYK. So, if treatment is provided against SYK it would be beneficial for Leishmaniasis. The study is predictable, as the authors themselves mentioned that “SFK inhibitors and Abl family kinase inhibitors decrease the uptake of Leishmania parasites (Wetzel et al., 2012; Wetzel et al., 2016). And Others have suggested that signaling during phagocytosis by these two kinase families can be relayed to SYK (Crowley et al., 1997; Greuber and Pendergast, 2012). The study is merely correlative and did not explore pathways regulated by SYK in phagocytosis, such as LAT (linker for activation of T cells), phosphatidylinositol 3-kinase (PI 3-K), and phospholipase Cγ (PLCγ). Neither they have shown which step of phagocytosis is specifically inhibited, as phagocytosis is a multi-step process. I had failed to discriminate between internalization and phagocytosis from their results. The other few concerns have been listed below.

1.SYK can be activated through different pathways .In some research articles it has been also demonstrated that adhesion molecule P-selectin glycoprotein ligand 1(PSGL1) found in cells associates with SYK and activates it. Experiments need to be performed in order to check the activity of PSGL1 in case of Leishmania infection. Valid explanation needs to be provided that why only Src-Abl-SYK pathway is being focused in this hypothesis.
2. As all the experiments need to performed atleast 3 times, error bar needs to be provided to all the graphical representation.
3. Fig 2A needs to be explained clearly.
4.Quantification needs to be provided properly for Fig1A and Fig2A.
5.As uptake of both promastigotes and amastigotes are shown, effect of concentration of entospletinib in promastigote uptake in RAW 264.7 also needs to be shown.
6. Effect of time in promastigote uptake while incubating with entospletinib needs to be shown.
7. Quantification of Fig 3A needs to be provided.
8. In Fig 5A the last lane of the blots are deformed compared to others. Replace the blot through fresh experiment.
9. In Fig 5 pSYK and pCrk is only seen in presence of amastigotes and not promastigotes. Clear explanation needs to be provided 11. A valid explanation is required why different cell lines are used.
12. The data for cytokine secretion could be represented graphically.
13. Grammatical error in line no 169 and 268.
14. Better schematic diagram could be provided for the hypothesis.
15. Line 183 states that Fig 2A and 2B represent the uptake of promastigote and amastigote which do not match with figure legend.

© 2023. Published by The Company of Biologists under the terms of the Creative Commons Attribution License (https://creativecommons.org/licenses/by/4.0/).

First revision

Author response to reviewers' comments

June 2, 2023

Response to review of JOCES/2022/260809, "The Src and Abl family kinases activate the Spleen Tyrosine Kinase to maximize phagocytosis and Leishmania infection"

Dear Dr. Billadeau and Colleagues:

We thank you for your supportive and helpful feedback on our manuscript. Our detailed, point-by-point responses to all of the issues raised by the referees and editorial office are attached along with our revised manuscript. As requested, all changes in the revised text have been highlighted in yellow. The line numbers indicated in this response refer to the highlighted pdf visible on the submission website, which was converted from the uploaded manuscript.

We believe that our responses to the reviewers' concerns have greatly strengthened the manuscript and our data. In particular, we have demonstrated the following:

1. We have clarified the mechanism by which SYK activation facilitates the internalization of *Leishmania* parasites, as requested by Reviewer 2. In particular, we did not find evidence that PSGL-1 binding or activation is required for SYK activity during amastigote uptake. We have also added additional controls for several of our experiments, as requested.

2. We have replaced our representative image in Figure 1A and made the revisions in the text requested by Reviewer 1.

3. We have made the editorial revisions requested.

After considering our responses to the reviewers' concerns and the additional data shown, we hope that you will find our manuscript to be acceptable for publication in the **Journal of Cell Science**. We look forward to your feedback.

Sincerely,
Dawn M. Wetzel, M.D., Ph.D.
Assistant Professor of Pediatrics and Biochemistry
UT Southwestern Medical Center

Editorial requests:
To the best of our knowledge, the paper now complies with all formatting guidelines. In particular:

1) We have revised the supplementary material so that it is now a single separate pdf.

2) We have provided a word file for the manuscript text, tables and legends, as well as a separate file for each figure.

3) We have reduced the length of the abstract so that it is under 180 words (edited between lines 45-8 on the highlighted manuscript converted to pdf format).

4) We have completed the submission checklist.

We are happy to make further revisions to meet any formatting guidelines that we have unintentionally overlooked. Please let us know if this is desired.

Reviewer 1

We thank Reviewer 1 for their supportive overview regarding our manuscript and have made the following revisions, as requested.

© 2023. Published by The Company of Biologists under the terms of the Creative Commons Attribution License (https://creativecommons.org/licenses/by/4.0/).

1) "Fig 1a. Why focus on a region where there are almost no beads in the middle of the FOV?"

We have replaced the top image in Figure 1A with another representative image.

2) "Fig 6b. It might help to note in line 754 that the parasite burden…was endpoint (day X)."

We have noted in the text and figure legend that the parasite burden was calculated at the termination of the experiment (week 18 for Figures 6A and B [text lines 316-7 on the highlighted manuscript after pdf conversion], and week 20 for Figures 6C and D [text line 327-8]).

3) "Tables 1 and 2 are relatively difficult to interpret without an unstimulated control. E.g. it is hard to know what 'low' means relative to 'high'. I am not getting much out of the cytokine data in the discussion – mostly because it is unclear what the homeostatic state was for these measures. I think it is fine to leave the data in, but maybe just note that more work would be needed to delineate differences?"

We apologize for our lack of clarity regarding the descriptions of the categories used in the cytokine data. We have added further information about "low" versus "high" stimulation, which refers to the number of *L. amazonensis* parasites contained in the lysate added for stimulation (lines 612-7 and Table 1 legend). We did run a negative (unstimulated) control in these assays to ensure that our values were not related to the homeostatic state, which we have clarified in the manuscript (lines 306-7). We have also noted in the discussion that further work is required to delineate changes in the immunological response to SYK inhibition in experimental murine leishmaniasis (lines 434-5).

4) "Line 319: "Our data add to the evidence that SYK is necessary for efficient C3bi-mediated phagocytosis." The density of compliment or IgG can make a huge difference…Maybe another way to say this is that 'our evidence supports Syk as a major contributor to efficient…" "

We agree with the reviewer and have changed this line to their suggested phrasing (line 363-4).

Reviewer 2

We thank the reviewer for their helpful recommendations, which have allowed us to improve our manuscript. We have performed the following experiments and have made the following edits, as requested. We have renumbered and combined Reviewer 2's points for clarity.

1. "SYK can be activated through different pathways…The study…did not explore pathways regulated by SYK in phagocytosis, such as LAT (linker for activation of T cells), phosphatidylinositol 3-kinase (PI 3-K), and phospholipase Cγ (PLCγ)…In some research articles it has been also demonstrated that adhesion molecule P-selectin glycoprotein ligand 1(PSGL1) found in cells associates with SYK and activates it. Experiments need to be performed in order to check the activity of PSGL1 in case of Leishmania infection. Valid explanation needs to be provided that why only Src-Abl-SYK pathway is being focused in this hypothesis." [Response to main paragraph and original comment #1.]

We completely agree with the reviewer that the pathways regulated by SYK during phagocytosis require further study. We have now included additional information about the current state of the evidence for the employment of the above-mentioned pathways in the discussion section (lines 368-72 in the highlighted manuscript that was converted to pdf by the journal website). A full characterization of all pathways potentially regulated by SYK during phagocytosis and *Leishmania* uptake is outside of the scope of this manuscript. Here, we have focused on delineating the next steps of the signaling pathway (Src/Abl) previously described by our group.

As requested, we explored whether *L. amazonensis* employs signaling by the adhesion molecule PSGL1 during uptake (lines 247-54). PSGL-1 has not been shown to be a receptor for *Leishmania* uptake, but it is known to activate SYK signaling via interactions with FcR. We explored both of these possibilities using an antibody known to block PSGL-1 signaling, which should also prevent *Leishmania* from directly interacting with PSGL-1. We found that this antibody did not affect

© 2023. Published by The Company of Biologists under the terms of the Creative Commons Attribution License (https://creativecommons.org/licenses/by/4.0/).

Leishmania internalization (Figure S3C). In addition, entospletinib still prevented *Leishmania* uptake when added alongside the antibody (Figure S3D). We are aware that a blocking antibody may not prevent signaling through the intracellular domain of PSGL-1, which we have commented on in the discussion section (lines 387-92). We are unable to identify a small molecule that blocks this PSGL-1 signaling directly (based on our literature review, the commercially-available inhibitors of selectins would also not prevent this signaling).

It is plausible that this possibility could be tested through genetic knockout of PSGL-1, but such studies are outside the scope of this manuscript.

2. "The authors have not shown which step of phagocytosis is specifically inhibited, as phagocytosis is a multi-step process. I had failed to discriminate between internalization and phagocytosis from their results." [Response to main paragraph].

We agree with the reviewer that phagocytosis is a multi-step process. SYK has previously been implicated in phagocytic cup closure. We have now clarified our manuscript in several areas to ensure that this point has been made (*e.g.,* lines 356-8 in the discussion section). A defect in cup closure is also supported by our data that despite differences seen in phagocytic indexes, there is no difference in the total number of phagocytic cups seen between DMSO and entospletinib-treated macrophages during amastigote internalization (Figure S3A).

In addition, we reviewed our images that included actin staining, and found that there is a significant difference in the intensity of actin fluorescence in phalloidin-labeled phagocytic cups between DMSO and entospletinib-treated macrophages that are internalizing *Leishmania* amastigotes, which suggests that SYK signaling is also affecting actin polymerization. We have now added these data as supplemental figures (Figures S2 and S3B) and have commented on it in the results section (lines 222-31) and the discussion (lines 356-8).

3. "As all the experiments need to performed at least 3 times, error bar needs to be provided to all the graphical representation." [Response to comment #2].

We apologize for the lack of clarity in our methods, which we have now corrected (lines 517-21). All the experiments shown were performed at least 3 times (3 biological replicates, which each contained multiple technical replicates). We normalize each of our biological replicates separately so that the control is set to 100% for each experiment. As such, there is no error bar shown for the control category. The experimental category then represents a percentage of this control value and has error bars. We use a one-sample *t*-test for statistical analysis of our results, which is the statistical method of choice when analyzing these types of data. This method has been previously published multiple times in the past by multiple groups, including by our group in *J Cell Science* (DOI: 10.1242/jcs.185595).

4. "Fig 2A needs to be explained clearly. Quantification needs to be provided properly for Fig 1A, Fig 2A, and 3A." [Original comments #3, 4, 7, and 15].

Figure 1A, 2A, and 3A are representative images for the quantified data in 1B, 2B, and 3B, respectively. For clarity and as requested, we have now commented on how many beads or parasites are internalized in each image in their respective figure legends. We have also clarified our description of Figure 2A and corrected the attribution error in original line #183.

5. "As uptake of both promastigotes and amastigotes are shown, effect of concentration of entospletinib in promastigote uptake in RAW 264.7 also needs to be shown." [Original comment #5].

This result has been added to Supplemental Figure 1 (Figure S1B). Interestingly, entospletinib's EC_{50} value for promastigote uptake appears to be higher than for amastigotes.

6. "Effect of time in promastigote uptake while incubating with entospletinib needs to be shown." [Original comment #6].

This result has been added to Supplemental Figure 1 (Figure S1F).

© 2023. Published by The Company of Biologists under the terms of the Creative Commons Attribution License (https://creativecommons.org/licenses/by/4.0/).

7. "In Fig 5A the last lane of the blots are deformed compared to others. Replace the blot through fresh experiment." [Original comment #8].

We have now provided all the blots that we quantified in Figure 5, as well as a full Western blot image for each experimental category (as suggested by *J Cell Science's* Best Practices for Authors), as supplemental data (Figure S4).

8. "In Fig 5 pSYK and pCrk is only seen in presence of amastigotes and not promastigotes. Clear explanation needs to be provided." [Original comment #9].

We only performed the experiment for amastigotes, and this is why promastigote data is not shown. We focused on amastigotes because this is the more biologically relevant life cycle stage, as it is the stage found in mammalian hosts (lines 277-8). We have also previously shown that Src family kinases are only responsible for amastigote and not promastigote uptake. Therefore inhibition of Src kinase signaling would only be expected to affect SYK phosphorylation in the presence of amastigotes, and not in promastigotes.

9. "A valid explanation is required why different cell lines are used." [Original comment #11; please note that there was no comment #10].

RAW 264.7 cells are a cell line that is infected with a murine leukemia virus. Because this virus could affect kinase signaling, we confirm our data with primary macrophages isolated from mice. We have clarified this point in the body of the manuscript (line 177-9).

10. "The data for cytokine secretion could be represented graphically." [Original comment #12].

We have added these data as graphs in supplemental figures, as requested. Figure S5A-E correspond to Table 1, and Figure S5F-I correspond to Table 2.

11. "A better schematic diagram could be provided for the hypothesis." [Original comment #14].

We have provided an alternate diagram for our hypothesis (Figure 6E).

12. "Grammatical error in line no 169 and 268." [Original comment #13].

We have corrected these errors. We also used this revision opportunity to check for additional errors in the original manuscript and have corrected any others that we have found.

<u>Second decision letter</u>

MS ID#: JOCES/2022/260809

MS TITLE: The Src and Abl family kinases activate the Spleen Tyrosine Kinase to maximize phagocytosis and Leishmania infection

AUTHORS: Imran Ullah, Umaru Barrie, Rebecca M Kernen, Emily T Mamula, Francis Tho Huu Khuong II, Laela M Booshehri, Emma L Rhodes, James M Bradford, Arani Datta, and Dawn M Wetzel
ARTICLE TYPE: Research Article

I am happy to tell you that your manuscript has been accepted for publication in Journal of Cell Science, pending standard ethics checks.

© 2023. Published by The Company of Biologists under the terms of the Creative Commons Attribution License (https://creativecommons.org/licenses/by/4.0/).

Reviewer 1

Advance summary and potential significance to field

I have read “The Src and Abl family kinases activate the Spleen Tyrosine Kinase to maximize phagocytosis and Leishmania infection” by Ullah et al. This paper describes a carefully conducted study illustrating the importance of Syk kinase of IgG-coated Leishmania amastigotes by macrophages. While not particularly surprising, the study is exceedingly well conducted using both knockout macrophages and inhibitors to delineate these effects. Moreover, it makes clear that an inhibitor of Syk under clinical investigation may be of some utility here. In addition, this work contributes to the understanding that Syk contributes to complement-mediated phagocytosis and the less well-understood concept that Abl1/2 contributes to both. Overall I believe that this paper will be of interest to the readership of JCS.

Comments for the author

All concerns addressed, I believe this paper is ready for publication in JCS.

Reviewer 2

Advance summary and potential significance to field

Revisions are satisfactory, the paper can be accepted.

Comments for the author

Revisions are satisfactory, the paper can be accepted.

© 2023. Published by The Company of Biologists under the terms of the Creative Commons Attribution License (https://creativecommons.org/licenses/by/4.0/).
